# Determinants of Chemical Pleurodesis Recurrence in Malignant Pleural Effusion: Mechanical Apposition or Systemic Inflammation? A Retrospective Cohort Study

**DOI:** 10.3390/medicina62091763

**Published:** 2026-09-13

**Authors:** Caner İşevi, Mehmet Gökhan Pirzirenli, Eren Yılmaz, Yavuz Karaca, Burçin Çelik, Yasemin Büyükkarabacak

**Affiliations:** 1 Department of Thoracic Surgery, Faculty of Medicine, Ondokuz Mayıs University, Atakum, 55139 Samsun, Turkey; mgpy@hotmail.com (M.G.P.); mderenyilmaz@gmail.com (E.Y.); cburcin@hotmail.com (B.Ç.); yaseminbuyukkarabacak@gmail.com (Y.B.); 2 Independent Researcher, Bahcelievler, 34192 Istanbul, Turkey; yavuzkaraca@gmail.com

**Keywords:** pleural effusion, malignant, pleurodesis, talc, propensity score, non-expandable lung, mediation analysis

## Abstract

*Background and Objectives:* Malignant pleural effusion (MPE) severely impairs quality of life; chemical pleurodesis is the standard palliative modality, but the relative contributions of systemic inflammation, tube caliber, and mechanical pleural apposition to failure remain controversial. This study evaluated independent determinants of pleurodesis recurrence, hospital length of stay, and 30-day mortality using causal inference and mediation modeling. *Materials and Methods:* We conducted a retrospective cohort study of 140 patients undergoing chemical pleurodesis for symptomatic MPE. Pre-procedural inflammatory biomarkers, including the Neutrophil-to-Lymphocyte Ratio (NLR), tube caliber, administration method (bedside slurry vs. Video-Assisted Thoracoscopic Surgery [VATS] poudrage), and lung re-expansion status were recorded. Multivariable logistic regression with nomogram construction, 1:1 propensity score matching (PSM), inverse probability of treatment weighting (IPTW), and bootstrap structural equation modeling (SEM) mediation analysis were performed. *Results:* Pleurodesis recurrence occurred in 28.6% (*n* = 40) and 30-day mortality in 16.4% (*n* = 23) of patients. Non-expandable lung (adjusted odds ratio [aOR] = 9.89, 95% CI: 3.98–26.29, *p* < 0.001) and suboptimal catheter position (aOR = 3.80, 95% CI: 1.11–14.84, *p* = 0.407) were the strongest independent predictors of recurrence (C-index = 0.851); non-expandable lung also increased mortality 5.21-fold (*p* = 0.036). Large-bore tubes reduced matched odds of catheter malposition (OR = 0.096) and recurrence (OR = 0.405, *p* = 0.493) in the propensity-matched cohort, and VATS poudrage shortened hospital stay by 6.40 days (*p* = 0.074) versus slurry after weighting. Exploratory mediation analysis showed NLR’s direct effect on recurrence was non-significant (β = 0.0147, *p* = 0.5897); although summed indirect pathways accounted for a numerically larger share of the total effect, the bootstrap confidence interval for the total indirect effect crossed zero (β = 0.0330, 95% CI: −0.0155 to 0.0742), with only the pathway via catheter malposition individually excluding zero (β = 0.0126, 95% CI: 0.0014–0.0416), suggesting a plausible but unconfirmed mechanical-mediation trend. Patients with no comorbidities unexpectedly had higher mortality than those with multiple comorbidities (25.0% vs. 7.1%, *p* = 0.19), reflecting younger age and greater cachexia (albumin 28.14 ± 4.54 g/L) rather than comorbidity itself. *Conclusions:* Pleurodesis efficacy in MPE appears to be governed primarily by mechanical visceroparietal apposition; systemic inflammation showed no significant direct effect on recurrence, and its possible indirect, mechanically mediated contribution should be regarded as hypothesis-generating pending prospective confirmation. Clinicians managing high-risk patients (elevated NLR, non-expandable lung, or cachexia) should prioritize mechanical optimization—large-bore drainage, VATS poudrage, or early indwelling pleural catheter placement.

## 1. Introduction

Malignant pleural effusion (MPE) is a severe oncological complication arising from direct pleural invasion or lymphatic obstruction by primary thoracic tumors (lung adenocarcinoma, malignant pleural mesothelioma) or extra-thoracic metastases (breast, gastrointestinal, gynecological) [1]. In this population, where median survival is limited to 3–12 months depending on etiology, progressive dyspnea and refractory respiratory failure severely impair quality of life [2]; accordingly, the central goal of MPE management is to achieve rapid symptomatic palliation while minimizing the morbidity of recurrent interventions [3].

Chemical pleurodesis—achieved by instilling sterile talc as a bedside suspension (talc slurry) or by VATS insufflation (talc poudrage)—has remained the gold-standard palliative modality for decades, aiming to induce a fibrino-collagenous symphysis between the visceral and parietal pleura [4]. Complete visceroparietal apposition of the lung parenchyma to the chest wall is a physiological prerequisite for this process [5].

Nevertheless, 20–35% of procedures fail, resulting in refractory recurrence and prolonged hospitalization [6]. The literature remains divided on the determinants of failure: BTS guidelines favor small-bore tubes based on limited early evidence [3], whereas the TIME1 randomized trial found small-bore tubes (12F) were associated with a higher pleurodesis recurrence rate than large-bore tubes (24F) [4], and further evidence suggests small tubes are prone to occlusion in fibrinous effusions, leading to suboptimal positioning and impaired lung expansion. Prognostic indices such as the LENT score further claim that pre-procedural systemic inflammation (NLR, CAR) and nutritional status (albumin) are directly correlated with failure and mortality [2].

However, most existing studies are limited to univariate/bivariate correlative analyses; whether systemic inflammation directly causes pleurodesis recurrence, or instead triggers an indirect mediation cascade—inducing fibrous septations and pleural thickening that lead to catheter malposition and non-expandable/trapped lung—has not been demonstrated biomathematically. Observational series are further limited by confounding by indication, as surgeons preferentially select patients with better performance status for VATS or large-bore tubes, obscuring the true causal superiority between methods. This selection bias has been empirically demonstrated in comparative pleurodesis cohorts, where survival and outcome differences attributable to treatment modality lost significance after adjustment for baseline performance status [7].

This study aims to examine, in a cohort of 140 patients undergoing chemical pleurodesis, the relationships among pre-procedural inflammatory/nutritional biomarkers, tube caliber, administration method, and mechanical expansion status, using multivariable logistic regression, 1:1 PSM, IPTW, and 5000-bootstrap SEM mediation analyses. The objective is to identify the confounder-adjusted independent determinants and pathophysiological mediation pathways underlying pleurodesis recurrence, hospital length of stay, and 30-day mortality. We hypothesized that the effect of systemic inflammation on pleurodesis recurrence is not direct, but is mediated through mechanical barriers such as catheter malposition and non-expandable lung.

## 2. Materials and Methods

### 2.1. Study Design, Ethical Approval, and Sample Selection

This study is a single-center, observational, analytical, retrospective cohort study examining the clinical, operative, radiological, and biochemical data of patients hospitalized for symptomatic malignant pleural effusion (MPE) who underwent palliative tube thoracostomy and chemical pleurodesis (talc sclerotherapy) between 1 January 2016, and 31 December 2025, at Ondokuz Mayıs University Faculty of Medicine, Department of Thoracic Surgery. The study protocol was conducted in full accordance with the principles of the Declaration of Helsinki (2013 revision) and was approved by the Ondokuz Mayıs University Clinical Research Ethics Committee (Approval number: 2025/16; date of approval: 28 January 2026; reference no. B.30.2.ODM.0.20.08/53). Informed consent for participation was not required as per local legislation (Law No. 6698 on the Protection of Personal Data, Republic of Turkey), given the retrospective, non-interventional design of the study and its exclusive reliance on de-identified data.

During the specified 10-year period, consecutive patients presenting with pleural effusion who had histopathologically confirmed malignancy (via positive pleural fluid cytology or pleural biopsy) were screened. Of 161 eligible cases, 21 patients were excluded due to incomplete pre-procedural biochemical data or loss to 90-day follow-up, yielding a final cohort of *N* = 140.

Inclusion criteria were: (1) age ≥ 18 years; (2) histopathologically or cytologically confirmed MPE; (3) drainage followed by chemical pleurodesis with sterile talc (slurry or poudrage) for symptomatic (progressive dyspnea) effusion; (4) complete pre-procedural complete blood count and serum biochemistry panels; (5) documented clinical and radiological follow-up data for at least 90 days post-procedure.

Exclusion criteria were: (1) non-malignant transudative/exudative effusions such as chylothorax, empyema, parapneumonic effusion, or tuberculosis (*n* = 7 patients with benign, non-MPE who underwent pleurodesis were retained in the cohort as a benign comparator subset for comparative etiological polarization analysis); (2) use of a non-talc sclerosing agent (tetracycline, bleomycin, povidone-iodine, etc.); (3) primary placement of an Indwelling Pleural Catheter (IPC) as initial treatment; (4) the 21 patients excluded for incomplete data or loss to follow-up described above. No rounding or truncation was applied to the dataset; all observations were analyzed at their absolute values.

### 2.2. Data Collection and Variable Standardization

Patients’ electronic medical records, surgical operative notes, bedside monitoring forms, and PACS radiological imaging archives were reviewed to construct a standardized case report form. Variables were standardized under three core domains:

- Demographic and Clinical Characteristics: Age (dichotomized as <65 vs. ≥65 years, and trichotomized as <60, 60–74, and ≥75 years), sex, effusion character (exudate/transudate, classified according to Light’s criteria), laterality (right/left hemithorax), and hospital length of stay (LOS, days). Chronic comorbidities were screened to derive an indexed comorbidity count (Comorbidity Count, 0–5) and a Comorbidity Burden category (None/Single/Multiple).
- Primary and Pathological Diagnosis Classification: Primary malignancy sites and histopathological subtypes were consolidated into 5 combined etiological groups to maximize statistical power and avoid sparse contingency table cells: (I) Thoracic malignancies (*n* = 58; comprising squamous cell carcinoma [*n* = 9], small cell/neuroendocrine carcinoma [*n* = 8], and predominantly primary lung adenocarcinoma [*n* = 41]; no cases of malignant pleural mesothelioma were identified in this cohort), (II) breast carcinoma (*n* = 22), (III) gastrointestinal/gynecological malignancies (*n* = 31), (IV) benign effusions (*n* = 7), and (V) other malignancies (*n* = 22).
- Inflammatory and Nutritional Biomarkers: Within 24–48 h pre-procedure, C-reactive protein (CRP, mg/L), serum albumin (g/L), hemoglobin (g/dL), and absolute neutrophil/lymphocyte/eosinophil counts (/µL) and percentages were obtained. The Neutrophil-to-Lymphocyte Ratio (NLR = absolute neutrophil count/absolute lymphocyte count) and C-Reactive Protein-to-Albumin Ratio (CAR = CRP/serum albumin) were calculated.
- Procedural and Mechanical Parameters: All patients received 4.00 g of intrapleural sterile, graded talc (Steritalc^®^, Novatech SA, Aubagne, France) per standard sclerotherapy protocol. Thoracostomy catheters were dichotomized as small-bore (≤16 Fr, consistent with BTS guidance [3]) or large-bore (≥20 Fr, within the size range shown to favor pleurodesis efficacy in randomized data [4]); this cutoff also reflects the natural bimodal distribution of tube sizes used in our cohort. Administration method was classified as bedside talc slurry (*n* = 126) or VATS talc poudrage (*n* = 14).

### 2.3. Mechanical and Radiological Outcome Definitions

Key radiological parameters assessing mechanical procedural success were evaluated by two independent thoracic surgeons using bedside posteroanterior (PA) chest radiographs and fluoroscopy/chest CT obtained 24–48 h post-pleurodesis:

- Catheter Position Optimality (Procedure Optimality): The position of the thoracostomy drain within the pleural cavity was assessed. Placement of the drain into the most anatomically dependent region of the pleural space (costophrenic recess/posterior basal sinus) with all side-holes freely draining within the pleural cavity was defined as optimal catheter position. Interlobar fissure entrapment, apical kinking/coiling, loculation within pleural septations with loss of drainage function, or parenchymal deviation of the catheter was classified as suboptimal catheter malposition.
- Lung Re-Expansion Status (Lung Expansion): Post-drainage lung parenchymal apposition to the chest wall was dichotomized as: (I) complete visceroparietal apposition (adequate re-expansion; *n* = 95)—complete apposition of the lung parenchyma to the chest wall with full obliteration of the intrapleural dead space; and (II) non-expandable lung (NEL)/trapped lung syndrome (inadequate re-expansion; *n* = 45)—failure of the lung to appose the chest wall due to loss of elastic recoil from visceral pleural thickening (pleural peel), malignant infiltration (lung entrapment), or endobronchial obstruction, resulting in a persistent residual pleural cavity (pneumothorax ex vacuo). Because catheter position and lung re-expansion were assessed 24–48 h after the procedure—after treatment had already been administered—these variables are more accurately characterized as early post-procedural predictors of the subsequent 90-day recurrence outcome, rather than pre-treatment baseline covariates, and are interpreted as such throughout this manuscript. Catheter position and lung re-expansion were assessed by consensus between the two thoracic surgeons rather than as independent parallel readings; therefore, no interobserver agreement statistic was calculated. Because this assessment occurred before the 90-day recurrence outcome had occurred, assessors could not have been influenced by knowledge of that outcome, although as treating surgeons they were not blinded to other clinical context.

### 2.4. Primary and Secondary Clinical Endpoints

- Primary endpoint—Pleurodesis recurrence: Defined as recurrence of symptomatic dyspnea, as documented in outpatient follow-up or readmission records, together with radiologically confirmed (PA chest radiograph, ultrasound, or chest CT) re-accumulation of ipsilateral pleural fluid within 90 days post-pleurodesis. Patients remaining asymptomatic and effusion-free throughout the 90-day period were classified as pleurodesis success (no recurrence).
- Secondary endpoint 1—Reintervention requirement (Reintervention Requirement): Performance of a second invasive intervention (repeat thoracentesis, secondary tube thoracostomy, IPC placement, or surgical decortication) for symptomatic palliation in patients with pleurodesis recurrence or refractory drainage. Reintervention was performed exclusively in the context of documented recurrence or refractory drainage in this cohort; no reinterventions were undertaken for unrelated indications.
- Secondary endpoint 2—30-day early mortality (Mortality 30 Day): All-cause mortality occurring within the first 30 calendar days following the pleurodesis procedure (deceased, *n* = 23), with surviving patients classified as alive (*n* = 117).
- Secondary endpoint 3—Procedural complication (Complication): Occurrence of major complications requiring additional medical/surgical intervention (severe hemorrhage, pneumonia, empyema, ARDS) during the index hospitalization or within the 90-day follow-up period, abstracted from electronic medical records, operative notes, and bedside monitoring forms. Minor, self-limited, and expected post-procedural effects (e.g., transient pain, low-grade fever) not requiring additional intervention were not systematically captured as a separate variable.

### 2.5. Statistical Analysis

All statistical analyses were performed using R statistical software (version 4.6.1, R Foundation for Statistical Computing, Vienna, Austria) with the RStudio (version 2026.6.0.242, Posit Software, PBC, Boston, MA, USA) interface. Descriptive statistics were reported first: normality of continuous variables was assessed via the Shapiro–Wilk test and visual diagnostics (Q-Q plots, histograms). Continuous variables were reported as mean ± SD and median (interquartile range), and categorical variables as absolute frequency (n) and percentage (%).

Univariate and bivariate inferential comparisons: Between-group differences in continuous variables were assessed using the non-parametric Mann–Whitney U test when normality assumptions were violated, or the independent-samples *t*-test (with Welch’s correction) for variables satisfying normality and Levene’s assumption (e.g., serum albumin). Comparisons across three or more groups (e.g., the 5-category primary diagnosis classification) used the Kruskal–Wallis H test or one-way ANOVA (F), with Bonferroni-corrected Dunn’s pairwise post-hoc testing (Z) for significant results. Associations between categorical variables were evaluated using Pearson’s chi-square (χ^2^) test or Monte Carlo-simulated Fisher’s exact test, depending on expected cell frequencies.

Advanced Analysis 1—Multivariable Logistic Regression and Nomogram Modeling: After screening for multicollinearity among candidate predictors identified as significant in univariate analysis or of established clinical relevance (variance inflation factor, VIF < 3.0), three separate multivariable logistic regression models were constructed for pleurodesis recurrence, 30-day mortality, and non-expandable lung. Adjusted odds ratios (aOR) and 95% confidence intervals (95% CI) were reported to reflect independent effects with other covariates held constant. Model discrimination was assessed via the area under the ROC curve (ROC-AUC/C-index); clinical nomograms for bedside risk prediction and 1000-bootstrap Hosmer–Lemeshow calibration curves were constructed using the *rms* package (version 8.1-1).

Advanced Analysis 2—Propensity Score Matching (PSM) and Inverse Probability of Treatment Weighting (IPTW): To address confounding by indication in this observational cohort, Inverse Probability of Treatment Weighting (IPTW, average treatment effect on the treated [ATT] estimand) was applied for administration method (VATS vs. slurry) using the WeightIt (version 1.7.0) and survey (version 4.5) packages, and 1:1 nearest-neighbor Propensity Score Matching (PSM, 0.20 SD caliper) was performed for tube caliber (large-bore vs. small-bore) using the MatchIt (version 4.7.2) package. Age, sex, albumin, hemoglobin, NLR, comorbidity burden, and primary diagnosis category were entered as baseline confounders in both balancing models. Covariate balance was confirmed via Love plots (standardized mean difference [SMD] < 0.10 threshold), and weighted/matched odds ratios (matched OR) were compared across the resulting pseudo-randomized cohorts.

Advanced Analysis 3—Structural Equation Modeling (SEM) Mediation Analysis: To test the proposed pathophysiological chain of systemic inflammation (NLR std) → trapped lung (Lung Expansion) → catheter malposition (Procedure Optimality) → pleurodesis recurrence (Recurrence), a Structural Equation Model was constructed using the lavaan package (version 0.6-21). NLR was standardized (z-score) to address scale heterogeneity, and a bias-corrected and accelerated (BCa) bootstrap simulation with 5000 replications was run to derive direct effect, indirect effect (via trapped lung/malposition), serial mediation, and total effect magnitudes with 95% bootstrap confidence intervals. The structural network was visualized as a high-contrast path diagram using the semPlot package (version 1.1.8).

For all analyses, a two-tailed significance threshold of *p* < 0.05 was adopted. Patients with missing data (*n* = 21) were excluded listwise prior to analysis; no imputation was performed on the final cohort of *N* = 140.

Finally, to address the potential etiological heterogeneity introduced by retaining the 7 patients with benign effusions (who were initially included to provide a baseline comparative reference), a sensitivity analysis was performed excluding these cases (N=133). The primary multivariable logistic regression models were re-executed on this strictly malignant cohort to determine whether the inclusion of non-malignant cases had unduly influenced the effect estimates for pleurodesis recurrence and 30-day mortality. The results of this sensitivity analysis are provided in Supplementary Table S2.

## 3. Results

### 3.1. Cohort Characteristics and Descriptive Statistics

Of the 161 eligible cases identified during the study period, 21 patients were excluded due to missing data or loss to follow-up, and the final analysis was conducted on *N* = 140 patients. The mean age of the cohort was 62.23 ± 13.14 years, with males comprising 55.0% (*n* = 77) and females 45.0% (*n* = 63) of the cases. The complete distribution of patients’ pre-procedural demographic, biochemical, procedural, and clinical outcome characteristics is presented in Table 1.

Of the procedural cohort, the majority underwent bedside talc slurry administration (90.0%, *n* = 126), with VATS talc poudrage performed in a smaller subset (10.0%, *n* = 14); large bore thoracostomy tubes (≥20 Fr) were used in 57.1% (*n* = 80) of cases and small-bore tubes (≤16 Fr) in 42.9% (*n* = 60). Pre-procedural inflammatory indices showed wide dispersion across the cohort (NLR, 9.51 ± 11.13; CAR, 2.65 ± 2.92). Over the 90-day follow-up period, chemical pleurodesis failed in 28.6% of patients (*n* = 40), and 30-day all-cause mortality was 16.4% (*n* = 23).

### 3.2. Sex-Based Comparative Analysis

**Figure 1:**
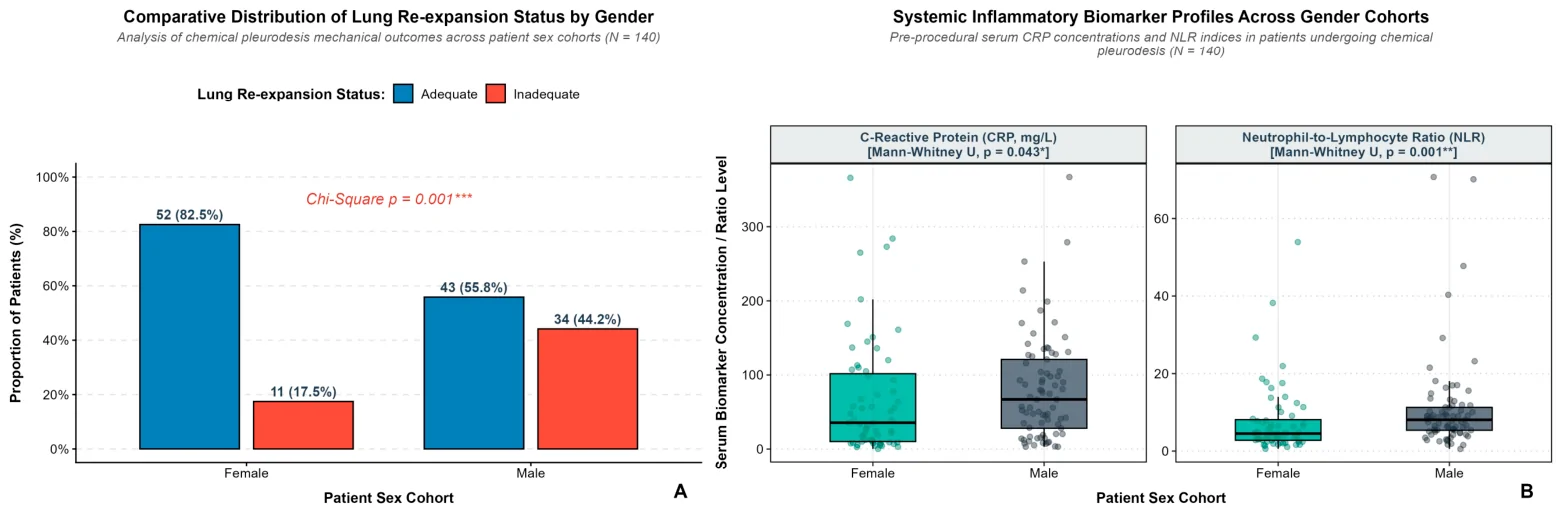
(**A**) Comparative distribution of lung re-expansion status by sex. (**B**) Systemic inflammatory biomarker profiles (CRP and NLR) across sex cohorts (*N* = 140). * *p* < 0.05; ** *p* < 0.01; *** *p* < 0.001.

Lung re-expansion status also differed markedly by sex: complete visceroparietal apposition was achieved in 82.5% of female patients compared with only 55.8% of male patients, while non-expandable lung/trapped lung was nearly 2.5-fold more frequent among males (44.2% vs. 17.5%; χ^2^(1) = 10.131, *p* = 0.01) (Figure 1A). Thoracic malignancies predominated among male patients (57.1%), whereas breast and gynecological malignancies were more frequent among female patients (31.7% and 19.0%, respectively); age distribution also differed significantly between sexes (χ^2^(2) = 6.016, *p* = 0.49).

Comparative analysis between sex cohorts also identified statistically significant differences (Mann–Whitney U or Pearson chi-square/Fisher’s exact tests, *p* < 0.05) in systemic inflammatory biomarkers. Male patients exhibited a significantly higher inflammatory burden than female patients, including higher C-reactive protein (80.82 ± 69.67 vs. 66.83 ± 78.91 mg/L, U = 1941.5, *p* = 0.43), neutrophil percentage (78.22% vs. 72.74%, *p* = 0.08), absolute neutrophil count (7726.14 vs. 5827.62/µL, *p* = 0.19), and Neutrophil-to-Lymphocyte Ratio (10.99 ± 12.42 vs. 7.70 ± 9.09, U = 1648.0, *p* = 0.01), alongside a correspondingly lower lymphocyte percentage (12.02% vs. 17.62%, *p* < 0.01) (Figure 1B).

### 3.3. Age-Based Comparative Analysis

**Figure 2:**
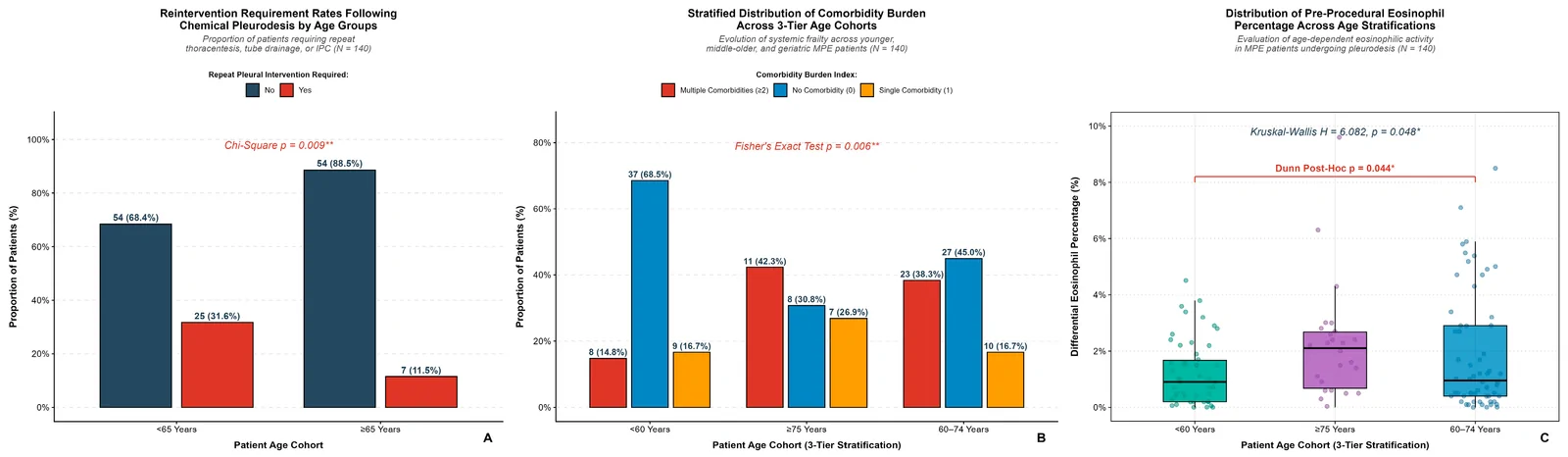
(**A**) Reintervention requirement rates by age cohort (<65 vs. ≥65 years). (**B**) Comorbidity burden distribution across 3-tier age strata (<60, 60–74, ≥75 years). (**C**) Pre-procedural eosinophil percentage across 3-tier age strata. * *p <* 0.05; ** *p <* 0.01.

When dichotomized at <65 versus ≥65 years, the two age groups were similar in most biochemical and procedural parameters but differed significantly in comorbidity burden, pleural fluid cytology, and reintervention requirement. Patients aged ≥ 65 years had a higher indexed comorbidity count than younger patients (1.28 ± 1.19 vs. 0.71 ± 1.22, U = 1668.0, *p* < 0.01). Patients aged < 65 years had a higher rate of positive pleural fluid cytology (67.1% vs. 50.8%, χ^2^(2) = 6.217, *p* = 0.28) and a higher reintervention requirement (31.6% vs. 11.5%, χ^2^(1) = 6.839, *p* = 0.09) (Figure 2A).

Refining the comparison into three age strata (<60, 60–74, and ≥75 years) demonstrated an increase in comorbidity burden with advancing age (Kruskal–Wallis H(2) = 13.387, *p* = 0.01; Dunn post-hoc <60 vs. 60–74, Z = 3.016, *p* = 0.08; <60 vs. ≥75, Z = 3.133, *p* = 0.05) (Figure 2B), together with differences in eosinophil percentage (H(2) = 6.082, *p* = 0.48; <60 vs. ≥75, Z = 2.438, *p* = 0.44) (Figure 2C) and tumor etiology (Fisher’s *p* = 0.49). Pleurodesis recurrence occurred in 33.3% of patients aged < 60 years and 19.2% of those aged ≥75 years.

### 3.4. Determinants of Pleurodesis Recurrence

When patients were stratified according to pleurodesis outcome (no recurrence, *n* = 100; recurrence, *n* = 40), pre-procedural inflammatory and nutritional biomarkers did not differ significantly between groups. CAR was 2.65 ± 3.05 in patients without recurrence and 2.66 ± 2.59 in those with recurrence, while serum albumin was 30.79 ± 7.09 and 30.85 ± 5.86 g/L, respectively (both *p* > 0.05). Significant between-group differences were identified for catheter position, lung re-expansion, chest tube diameter, and hospital length of stay (Table 2).

**Figure 3:**
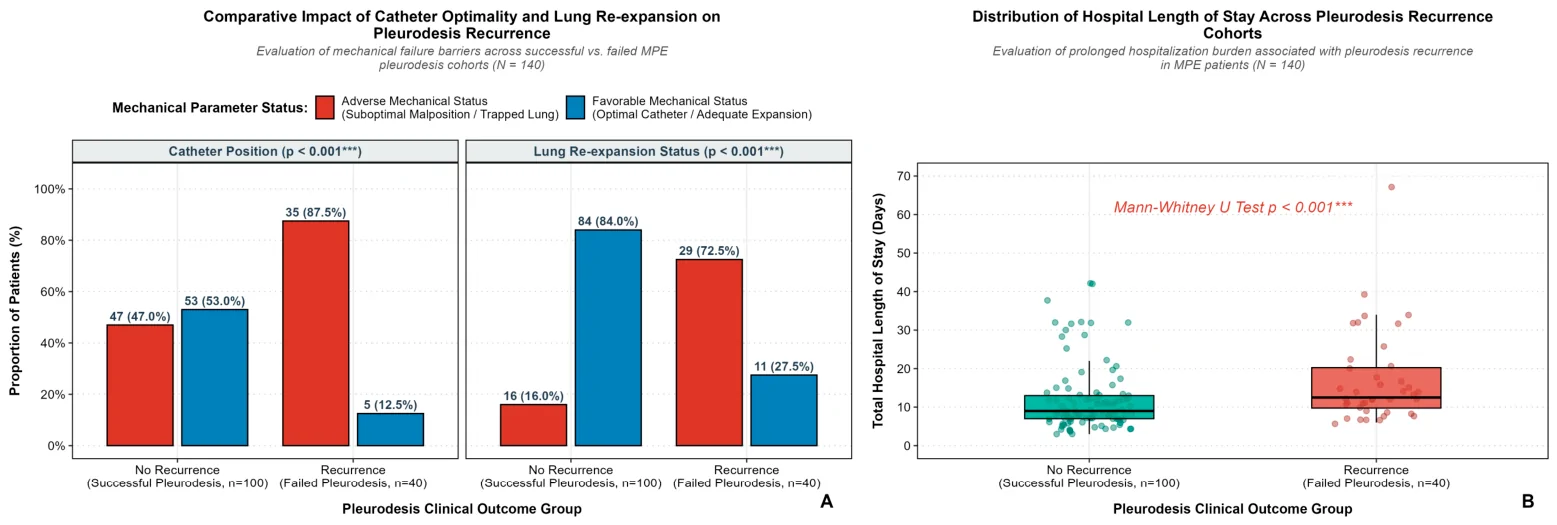
(**A**) Comparative impact of catheter optimality and lung re-expansion status on pleurodesis recurrence. (**B**) Distribution of hospital length of stay across pleurodesis recurrence cohorts (*N* = 140). *** *p* < 0.001.

Suboptimal catheter position was present in 87.5% of patients with recurrence compared with 47.0% of those without recurrence (χ^2^(1) = 17.680, *p* < 0.01) (Figure 3A). Inadequate lung re-expansion was observed in 72.5% of the recurrence group versus 16.0% of the non-recurrence group (χ^2^(1) = 39.266, *p* < 0.01) (Figure 3A). Patients with recurrence also had a smaller mean chest tube diameter (21.02 ± 9.72 vs. 24.32 ± 9.11 Fr, U = 2394.0, *p* = 0.48) and a longer hospital stay (17.12 ± 12.02 vs. 12.07 ± 8.79 days, U = 1265.0, *p* < 0.01) (Figure 3B). Reintervention was required in 80.0% of patients with recurrence compared with none of those without recurrence (χ^2^(1) = 99.216, *p* < 0.01), while 30-day mortality was 35.0% versus 9.0%, respectively (χ^2^(1) = 12.238, *p* < 0.01).

### 3.5. Determinants of Catheter Position Optimality

**Figure 4:**
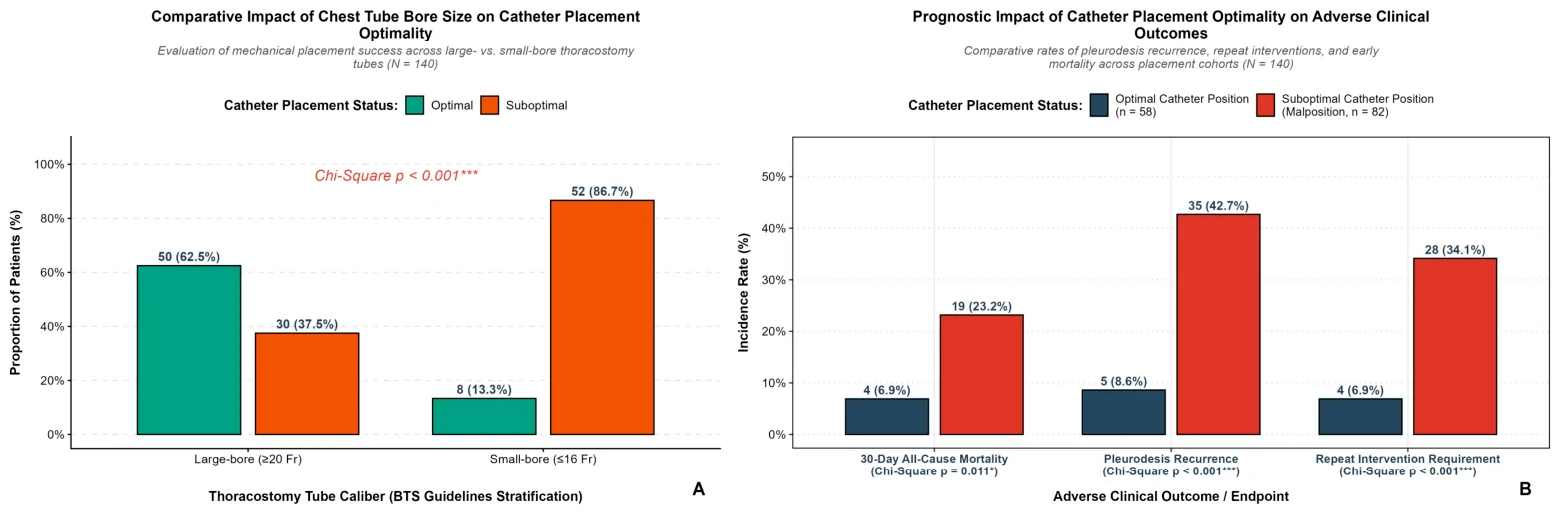
(**A**) Comparative impact of chest tube bore size on catheter placement optimality. (**B**) Prognostic impact of catheter placement optimality on adverse clinical outcomes (*N* = 140). * *p* < 0.05; *** *p* < 0.001.

Factors associated with optimal and suboptimal catheter position were subsequently examined. Catheter position differed significantly according to tube caliber and administration method (Table 3). Optimal positioning was achieved in 86.2% of large-bore tube cases compared with 36.6% of small-bore tube cases (χ^2^(1) = 32.160, *p* < 0.01) (Figure 4A), and all VATS talc poudrage cases had optimal catheter placement (14/14), compared with 75.9% of patients with optimal positioning in the bedside slurry group (χ^2^(1) = 19.392, *p* < 0.01). Adequate lung re-expansion was observed in 87.9% of patients with optimal catheter position compared with 53.7% of those with suboptimal position (χ^2^(1) = 16.757, *p* < 0.01).

Patients with suboptimal catheter position had a higher neutrophil percentage (77.31 ± 12.39% vs. 73.56 ± 11.50%, *p* = 0.25), lower lymphocyte percentage (13.07 ± 10.40% vs. 16.62 ± 9.01%, *p* = 0.02), and higher NLR (11.11 ± 12.65 vs. 7.24 ± 8.11, U = 1705.0, *p* = 0.04) than those with optimal positioning. Suboptimal catheter position was also associated with higher pleurodesis recurrence (42.7% vs. 8.6%, *p* < 0.01), reintervention requirement (34.1% vs. 6.9%, *p* < 0.01), and 30-day mortality (23.2% vs. 6.9%, *p* = 0.11) (Figure 4B).

### 3.6. Administration Method: Talc Slurry vs. Vats Poudrage

Comparison of administration methods showed significant differences between bedside talc slurry (*n* = 126) and VATS talc poudrage (*n* = 14). Optimal catheter position was observed in 100% of VATS cases compared with 34.9% of slurry cases (χ^2^(1) = 19.392, *p* < 0.01), and large-bore tubes were used in 100% versus 52.4%, respectively (χ^2^(1) = 9.803, *p* = 0.02). Hospital length of stay was shorter in the VATS group (6.93 ± 1.64 vs. 14.25 ± 10.31 days, U = 1382.5, *p* < 0.01). Patients undergoing VATS also had higher serum albumin (35.85 ± 11.22 vs. 30.25 ± 5.86 g/L, *p* = 0.01), higher hemoglobin (*p* = 0.16), higher absolute lymphocyte counts (*p* < 0.01), and lower NLR (4.21 ± 3.00 vs. 10.10 ± 11.55, *p* = 0.02).

### 3.7. Chest Tube Caliber: Large-Bore vs. Small-Bore

Large-bore tubes (≥20 Fr, *n* = 80) were associated with optimal catheter position in 62.5% of cases compared with 13.3% of small-bore tube cases (≤16 Fr, *n* = 60) (χ^2^(1) = 32.160, *p* < 0.01). All 14 VATS poudrage procedures used large-bore tubes, whereas small-bore tubes were used exclusively with bedside slurry (χ^2^(1) = 9.803, *p* = 0.02). Thoracic malignancies were more frequent among patients receiving small-bore tubes (51.7% vs. 33.8%, Fisher’s *p* = 0.49). Baseline NLR, CAR, and albumin did not differ significantly between tube-caliber groups (all *p* > 0.05). Pleurodesis recurrence (36.7% vs. 22.5%), reintervention (26.7% vs. 20.0%), and 30-day mortality (20.0% vs. 13.8%) were numerically higher in the small-bore group, although these unadjusted differences were not statistically significant.

### 3.8. The Comorbidity-Mortality Discrepancy

**Figure 5:**
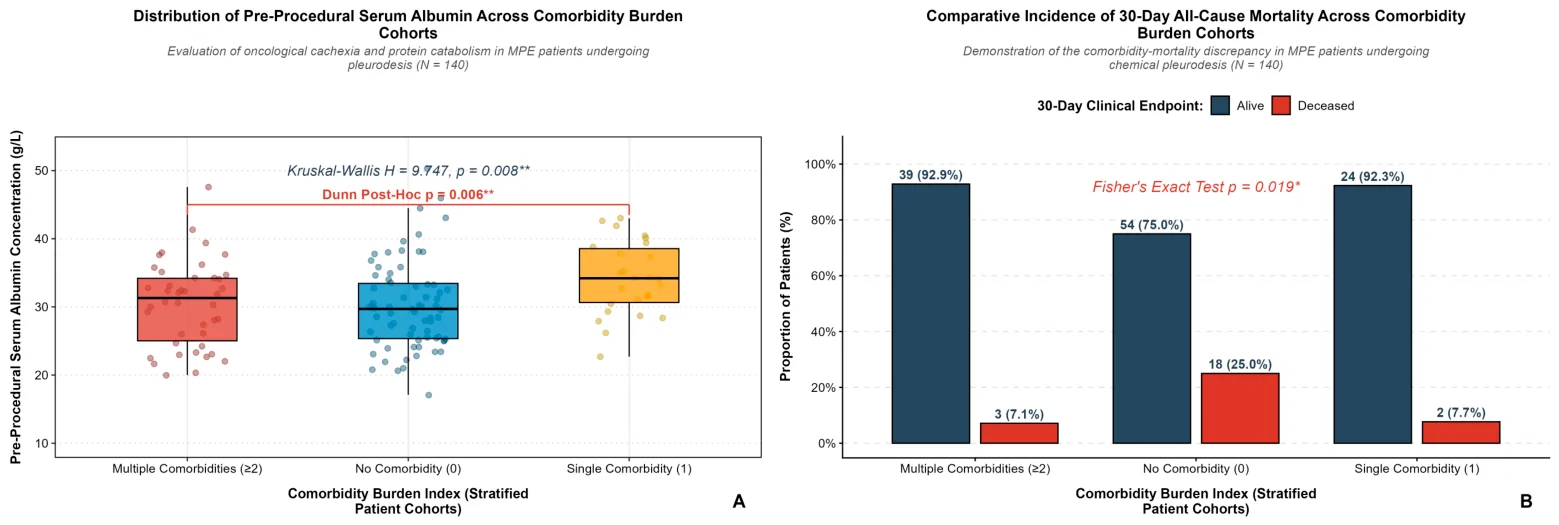
(**A**) Distribution of pre-procedural serum albumin across comorbidity burden cohorts. (**B**) Comparative incidence of 30-day all-cause mortality across comorbidity burden cohorts, demonstrating the comorbidity-mortality discrepancy (*N* = 140). * *p <* 0.05; ** *p* < 0.01.

Patients without indexed comorbidities were younger than those with single or multiple comorbidities (57.92 ± 13.08 vs. 64.92 ± 12.44 and 67.95 ± 11.12 years, respectively; F(2,137) = 9.435, *p* < 0.01). Serum albumin also differed among groups (H(2) = 9.747, *p* = 0.08), with lower levels in patients without comorbidities than in those with a single comorbidity (29.80 ± 7.15 vs. 34.16 ± 5.35 g/L, Dunn post-hoc *p* = 0.06) (Figure 5A).

Patients were stratified according to comorbidity burden as no comorbidity (*n* = 72), single comorbidity (*n* = 26), or multiple comorbidities (≥2; *n* = 42). Thirty-day all-cause mortality differed significantly among these groups, occurring in 25.0% of patients without indexed comorbidities, 7.7% of those with a single comorbidity, and 7.1% of those with multiple comorbidities (Fisher’s *p* = 0.19) (Figure 5B). Administration method, tube caliber, and catheter position did not differ significantly across comorbidity groups (all *p* > 0.05).

**Note.** Age was compared using one-way ANOVA; serum albumin was compared using the Kruskal–Wallis H test with Bonferroni-corrected Dunn post-hoc testing; 30-day mortality was compared using Fisher’s exact test. Only variables reaching statistical significance (*p* < 0.05) are shown.

### 3.9. Laterality: Right vs. Left Hemithorax

Right-sided (*n* = 85) and left-sided (*n* = 55) interventions showed no significant differences in catheter position, lung re-expansion, tube caliber, administration method, hospital length of stay, pleurodesis recurrence, reintervention requirement, or 30-day mortality (all *p* > 0.05). Right-sided effusions were associated with higher neutrophil percentage (77.83 ± 10.58% vs. 72.55 ± 13.69%, *p* = 0.25) and NLR (10.21 ± 10.79 vs. 8.42 ± 11.66, *p* = 0.38). Tumor distribution also differed by laterality, with thoracic malignancies occurring more frequently on the right (49.4% vs. 29.1%) and breast carcinoma more frequently on the left (23.6% vs. 10.6%; Fisher’s *p* = 0.48).

### 3.10. Systemic Inflammation as a Driver of Lung Re-Expansion Failure

**Figure 6:**
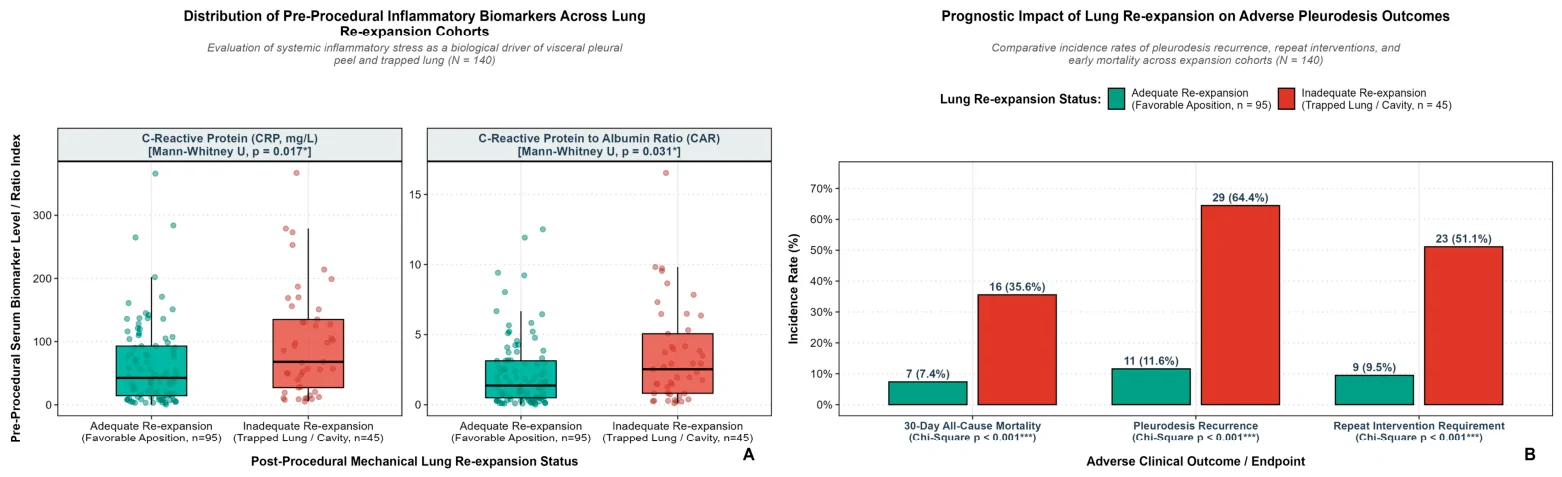
(**A**) Distribution of pre-procedural inflammatory biomarkers (CRP and CAR) across lung re-expansion cohorts. (**B**) Prognostic impact of lung re-expansion status on adverse pleurodesis outcomes (*N* = 140). * *p* < 0.05; *** *p* < 0.001.

Pre-procedural characteristics were compared between patients with adequate lung re-expansion (*n* = 95) and those with inadequate re-expansion/non-expandable lung (*n* = 45) (Table 4). Patients with inadequate re-expansion had higher CRP (97.24 ± 85.92 vs. 63.76 ± 65.45 mg/L, U = 1599.5, *p* = 0.17), absolute neutrophil count (7792.22 ± 4006.29 vs. 6435.82 ± 4512.05/µL, U = 1696.0, *p* = 0.49), and CAR (3.50 ± 3.48 vs. 2.25 ± 2.54, U = 1652.0, *p* = 0.31) (Figure 6A). Male sex was more frequent among patients with inadequate re-expansion (75.6% vs. 45.3%, χ^2^(1) = 10.131, *p* = 0.01).

Suboptimal catheter position was observed in 84.4% of patients with inadequate re-expansion compared with 46.3% of those with adequate re-expansion (χ^2^(1) = 16.757, *p* < 0.01). Inadequate re-expansion was also associated with longer hospital stay (16.36 ± 11.39 vs. 12.17 ± 9.09 days, *p* = 0.01), higher pleurodesis recurrence (64.4% vs. 11.6%, χ^2^(1) = 39.266, *p* < 0.01) (Figure 6B), higher reintervention requirement (51.1% vs. 9.5%, *p* < 0.01), and higher 30-day mortality (35.6% vs. 7.4%, *p* < 0.01).

### 3.11. Determinants of 30-Day Mortality

**Figure 7:**
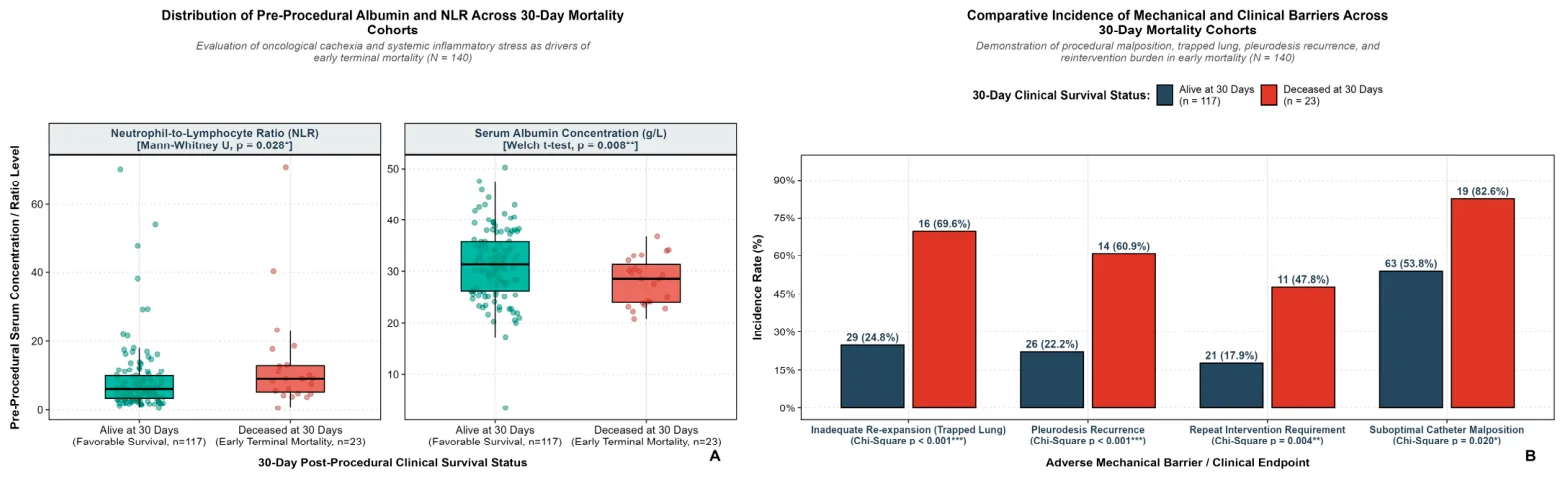
(**A**) Distribution of pre-procedural serum albumin and NLR across 30-day mortality cohorts. (**B**) Comparative incidence of mechanical and clinical failure barriers (trapped lung, pleurodesis recurrence, reintervention, catheter malposition) across 30-day mortality cohorts (*N* = 140). * *p* < 0.05; ** *p* < 0.01; *** *p* < 0.001.

Among the 140 patients, 117 were alive and 23 had died within 30 days of pleurodesis (Table 5)**.** Patients who died had a lower indexed comorbidity count (0.61 ± 1.50 vs. 1.03 ± 1.17, U = 1750.5, *p* = 0.14) and lower serum albumin (28.14 ± 4.54 vs. 31.33 ± 6.99 g/L, t(45.43) = 2.785, *p* = 0.08) (Figure 7A). They also had higher NLR (13.24 ± 15.06 vs. 8.78 ± 10.11, *p* = 0.28) and neutrophil percentage (80.50 ± 12.54% vs. 74.82 ± 11.88%, *p* = 0.12), together with lower lymphocyte (11.89 ± 11.51% vs. 15.06 ± 9.60%, *p* = 0.37) and eosinophil percentages (0.93 ± 1.12% vs. 1.82 ± 1.91%, *p* = 0.27) (Figure 7A).

Suboptimal catheter position was more frequent among patients who died within 30 days (82.6% vs. 53.8%, χ^2^(1) = 5.421, *p* = 0.20), as was inadequate lung re-expansion (69.6% vs. 24.8%, χ^2^(1) = 15.677, *p* < 0.01) (Figure 7B). Patients who died also had longer hospital stays (21.26 ± 10.05 vs. 11.99 ± 9.35 days, *p* < 0.01), higher pleurodesis recurrence (60.9% vs. 22.2%, *p* < 0.01), and higher reintervention rates (47.8% vs. 17.9%, *p* = 0.04).

### 3.12. Determinants of Reintervention Requirement

Among 140 patients, 32 required a repeat invasive intervention. Suboptimal catheter position was present in 87.5% of patients requiring reintervention compared with 50.0% of those who did not (χ^2^(1) = 12.802, *p* < 0.01), while inadequate lung re-expansion was present in 71.9% versus 20.4%, respectively (χ^2^(1) = 27.708, *p* < 0.01). All patients requiring reintervention had documented pleurodesis recurrence (χ^2^(1) = 99.216, *p* < 0.01). Patients requiring reintervention were younger (57.59 ± 13.89 vs. 63.60 ± 12.65 years, U = 2125.5, *p* = 0.49), and 78.1% were aged <65 years compared with 50.0% of patients without reintervention (χ^2^(1) = 6.839, *p* = 0.09). Reintervention was also associated with longer hospital stay (17.38 ± 12.82 vs. 12.37 ± 8.80 days, U = 1150.0, *p* = 0.04) and higher 30-day mortality (34.4% vs. 11.1%, χ^2^(1) = 8.110, *p* = 0.04).

### 3.13. Multivariable Predictors of Clinical Outcomes

Three multivariable logistic regression models were constructed for pleurodesis recurrence, 30-day mortality, and non-expandable lung (Table 6). In Model 1 (recurrence, ROC-AUC = 0.851), inadequate lung re-expansion was the strongest independent predictor (aOR = 9.89, 95% CI: 3.98–26.29, *p* < 0.001), followed by suboptimal catheter position (aOR = 3.80, 95% CI: 1.11–14.84, *p* = 0.407). NLR (aOR = 1.01, *p* = 0.6794), chest tube caliber (aOR = 1.25, *p* = 0.6779), and age (aOR = 0.97, *p* = 0.1586) were not independently associated with recurrence after adjustment.

In Model 2 (30-day mortality, ROC-AUC = 0.825), inadequate lung re-expansion was independently associated with mortality (aOR = 5.21, 95% CI: 1.77–16.74, *p* = 0.036). Serum albumin did not reach statistical significance after adjustment (aOR = 0.93, *p* = 0.893), and catheter position, NLR, eosinophil percentage, and comorbidity count were also not independently associated with mortality. In Model 3 (non-expandable lung), male sex was the only variable independently associated with inadequate re-expansion (aOR = 3.67, 95% CI: 1.65–8.70, *p* = 0.020), whereas CRP, CAR, NLR, and tube caliber were not significant. Clinical nomograms based on Models 1 and 2 are shown in Supplementary Figures S1 and S2.

### 3.14. Causal Inference: Psm and Iptw Analyses

To address measured baseline differences in administration method and tube caliber, IPTW and 1:1 PSM were applied using age, sex, albumin, hemoglobin, NLR, comorbidity burden, and primary diagnosis category as covariates (Table 7). After IPTW, all covariate standardized mean differences were below 0.10 (Supplementary Figure S3). In the weighted analysis, VATS talc poudrage was associated with a 6.40-day shorter hospital stay than bedside slurry (*p* = 0.074) and markedly lower odds of suboptimal catheter placement (OR < 0.001, *p* < 0.001). The weighted odds ratio for pleurodesis recurrence was 0.246 but did not reach statistical significance (*p* = 0.2057).

After 1:1 PSM with a 0.20-SD caliper, 98 patients were retained in the matched cohort, with covariate balance meeting the predefined threshold across measured covariates (Supplementary Figure S4). Large-bore tube use was associated with lower odds of catheter malposition (matched OR = 0.096, *p* < 0.001) and pleurodesis recurrence (matched OR = 0.405, *p* = 0.493). The matched odds ratio for non-expandable lung was 0.457 and did not reach statistical significance.

### 3.15. Structural Equation Modeling: Mediation Analysis (Supplementary Materials)

A structural equation model was constructed to evaluate the association between standardized NLR and pleurodesis recurrence through inadequate lung re-expansion and catheter malposition, using 5000 BCa bootstrap replications (Supplementary Table S1). The direct effect of NLR on recurrence was not statistically significant (β = 0.0147, 95% CI: −0.0472 to 0.0615, *p* = 0.5897). Among the individual indirect pathways, only the pathway through catheter malposition had a bootstrap confidence interval excluding zero (β = 0.0126, 95% CI: 0.0014 to 0.0416). The indirect pathway through inadequate re-expansion (β = 0.0178, 95% CI: −0.0224 to 0.0550) and the serial pathway through inadequate re-expansion and catheter malposition (β = 0.0026, 95% CI: −0.0023 to 0.0110) had confidence intervals including zero.

The total indirect effect was β = 0.0330 (95% CI −0.0155 to 0.0742), corresponding numerically to 69.3% of the total estimated effect (0.0330/0.0476); however, the bootstrap confidence interval for the total indirect effect included zero. The total combined effect was β = 0.0476 (95% CI: −0.0409 to 0.1142). The estimated structural pathways are illustrated in Supplementary Figure S5.

### 3.16. Sensitivity Analysis in the Restricted Malignant Cohort

To ensure that our primary findings were not driven by the inclusion of the 7 patients with benign effusions, a sensitivity analysis was conducted on the restricted, MPE-only cohort (N=133). When the multivariable logistic regression models were re-run on this homogeneous subset, the effect estimates for the primary mechanical predictors remained consistent with the main analysis. Specifically, inadequate lung expansion continued to show a strong independent association with both pleurodesis recurrence (aOR = 9.65, 95% CI: 3.81–26.10, p<0.0001) and 30-day mortality (aOR = 4.49, 95% CI: 1.48–14.80, p=0.0098). Suboptimal catheter position also retained its independent association with recurrence (aOR = 4.43, 95% CI: 1.22–19.20, p=0.0315). Consistent with the primary models, baseline systemic inflammatory markers (e.g., NLR) and chronological age did not reach independent statistical significance in this restricted cohort. These findings (detailed in Supplementary Table S2) support the observation that the relationship between mechanical failure and adverse pleurodesis outcomes is stable and persists even when non-malignant cases are excluded from the analysis.

## 4. Discussion

### 4.1. Principal Findings

In this retrospective cohort of 140 patients undergoing chemical pleurodesis, inadequate lung re-expansion and suboptimal catheter position were the principal independent predictors of pleurodesis recurrence, whereas baseline NLR was not independently associated with recurrence after multivariable adjustment. Propensity-adjusted analyses showed persistent associations between large-bore tube use and favorable mechanical outcomes and between VATS poudrage and shorter hospitalization after adjustment for measured baseline differences. In exploratory SEM analysis, the direct association between NLR and recurrence was not significant, while catheter malposition was the only individual indirect pathway with a bootstrap confidence interval excluding zero. Collectively, these findings suggest that adequate mechanical pleural apposition may be more closely related to pleurodesis outcome than baseline systemic inflammatory status, while a potential indirect relationship between inflammation and mechanical failure warrants further prospective investigation.

### 4.2. Mechanical Determinants: Concordance with the Existing Literature

The centrality of mechanical apposition to pleurodesis success is well established. A systematic review and meta-analysis of 34 studies identified full lung re-expansion after drainage and smaller pre-pleurodesis effusion volume as the strongest predictors of success [8], a finding that our multivariable model reproduces and substantially extends by quantifying the failure risk attributable to incomplete re-expansion (aOR = 9.89). Higher pleural fluid pH has also been repeatedly identified as a strong predictor of success, likely reflecting both more favorable mechanical conditions and less extensive pleural disease burden [9,10,11], and lower pleural glucose and higher LDH have shown a similar, if more heterogeneous, association with failure [8,9]. In a cohort of over 1000 tunneled pleural catheter placements, hydropneumothorax with incomplete lung expansion independently reduced pleurodesis rates, a mechanistic parallel to our own finding that catheter malposition—the anatomical precursor of incomplete apposition—nearly quadrupled the odds of failure. Interestingly, crude comparisons of recurrence according to tube caliber were not statistically significant despite the strong association between tube caliber and catheter positioning. This divergence suggests that tube caliber may be relevant primarily through its relationship with effective drainage and catheter positioning rather than as an independent determinant of pleurodesis biology. Our PSM analysis adds a causal-inference framing to this literature: within a matched pseudo-cohort balanced for age, sex, albumin, hemoglobin, NLR, comorbidity burden, and diagnosis, large-bore tubes were associated with over 90% lower matched odds of malposition and nearly 60% lower matched odds of recurrence. As in any observational analysis, PSM can only adjust for measured confounders, and residual confounding by indication or unmeasured factors (e.g., performance status, operator technique) cannot be excluded; this finding should therefore be regarded as preliminary evidence consistent with a possible causal contribution of tube caliber to mechanical failure, rather than definitive proof of superiority.

The strong coexistence of catheter malposition and inadequate lung re-expansion underscores the close mechanical interdependence between effective pleural drainage and visceroparietal apposition. We report a moderate correlation between the two (φ ≈ 0.35, derived from χ^2^(1) = 16.757, *N* = 140, consistent with our multicollinearity screening (VIF < 3.0), which supported modeling both as distinct predictors. Given the single-timepoint radiographic assessment, our design cannot definitively establish the direction of this relationship; we consider it likely that both mechanical findings are downstream manifestations of a shared underlying process—fibrinous pleural septation and visceral pleural thickening—rather than a simple unidirectional cause-effect relationship, consistent with our proposed mediation framework.

The clinical relevance of these mechanical factors extended beyond the recurrence endpoint. Patients requiring reintervention showed the same pattern of catheter malposition and inadequate lung re-expansion, suggesting that failure to achieve adequate mechanical conditions at the index procedure is closely associated with the subsequent need for additional invasive palliation.

### 4.3. Systemic Inflammation as an Indirect, Mediated Pathway

The role of systemic inflammation in pleurodesis outcome has been demonstrated primarily through correlative designs. In the TIME1 trial dataset, a significantly greater rise in serum CRP distinguished successful from failed pleurodesis (mean difference 19.2 mg/L, 95% CI 6.2–32.0, *p* = 0.04), while white cell count change did not differ between groups [12], and a separate retrospective cohort confirmed CRP change as an independent predictor of efficacy [13]. Pleural fluid CRP ≥ 30 mg/L predicted success in 90% of patients in a small prospective series [14], and cytokine profiling has shown that an early IL-10 increment within three hours characterizes successful pleurodesis, whereas failure is associated with delayed TNF-α and IL-10 rises at 3–24 h [15]. More recently, lower pleural sRAGE has been linked to talc pleurodesis recurrence, suggesting an imbalance between sRAGE and HMGB1 signaling in unsuccessful symphysis [16].

None of these studies, however, formally tested whether inflammation acts on pleurodesis outcome directly or through an intermediate structural pathway. In our cohort, NLR was significantly higher among patients with suboptimal catheter positioning despite showing no independent association with recurrence in the final multivariable model. This pattern is compatible with the possibility that systemic inflammatory burden is associated with pleural conditions that predispose to technical drainage failure rather than exerting a direct effect on pleurodesis efficacy.

The association between higher CRP/CAR and inadequate lung re-expansion provides additional support for a relationship between inflammatory burden and the mechanical phenotype. However, pleural fibrosis, septation, visceral pleural thickness, and loculation were not directly quantified in this cohort. Accordingly, these findings should be interpreted as evidence of association with non-expandable lung rather than proof that systemic inflammation directly induces fibrotic pleural restriction.

Our SEM analysis provides exploratory support for this framework. The direct association between standardized NLR and recurrence was not statistically significant, whereas the indirect pathway through catheter malposition was the only individual pathway whose BCa bootstrap confidence interval excluded zero. Although the summed indirect effects numerically represented approximately 69% of the combined estimated effect, the confidence interval for the total indirect effect included zero. The mediation findings should therefore be interpreted as hypothesis-generating rather than as definitive evidence of a causal pathway.

The observed pattern is compatible with the hypothesis that systemic inflammatory burden contributes indirectly to pleurodesis recurrence through mechanical intermediates, particularly catheter malposition. Inflammatory pleural remodeling, fibrin deposition, loculation, and visceral pleural restriction represent biologically plausible mechanisms through which this association might arise [5]; however, these structural processes were not directly measured in the present cohort and therefore should be regarded as potential mechanisms rather than demonstrated mediators. This framework may also help explain why prognostic indices incorporating inflammatory markers, such as the LENT score [2], retain prognostic value even when baseline inflammatory biomarkers do not independently predict pleurodesis recurrence after adjustment for mechanical variables.

### 4.4. Sex and Cancer Type as Upstream Determinants of Mechanical Failure

A further upstream determinant of mechanical failure emerged unexpectedly from our data: male sex. Male patients had a nearly 2.5-fold higher rate of non-expandable lung than female patients (44.2% vs. 17.5%), paralleled by a higher inflammatory burden (NLR 10.99 ± 12.42 vs. 7.70 ± 9.09) and a predominance of thoracic malignancy (57.1% of male patients, versus breast and gynecological malignancies in 50.7% of female patients). The marked sex difference in lung re-expansion occurred alongside substantial differences in tumor distribution, with thoracic malignancies predominating among men and breast/gynecological primaries among women. This raises the possibility that the observed sex effect partly reflects differences in underlying tumor biology rather than sex alone.

In the multivariable model for non-expandable lung, male sex was in fact the only variable that retained independent significance (aOR = 3.67, 95% CI: 1.65–8.70, *p* = 0.020); CRP, CAR, and NLR each lost significance once sex was accounted for. The persistence of male sex, but not CRP, CAR, or NLR, in the adjusted model suggests that sex may capture differences not fully represented by these inflammatory biomarkers, including tumor distribution, extent of pleural involvement, or other unmeasured biological factors. Whether this reflects sex-linked differences in cytokine signaling, in pleural mesothelial biology, or simply in the distribution of thoracic versus breast/gynecological primaries in our cohort cannot be resolved from an observational design, but the finding argues that risk stratification for trapped lung should consider sex alongside inflammatory markers rather than relying on the latter alone.

Not every observation in the literature fits a purely mechanical framework. Mesothelioma has been associated with a significantly lower pleurodesis success rate than other malignancies (73.3% vs. 84.9%, *p* = 0.23) despite mounting an equivalent systemic inflammatory response, suggesting that impaired local pleural inflammation from disrupted mesothelial architecture—rather than incomplete apposition alone—may drive failure in this subgroup [12]. Cancer type more broadly has been shown to influence outcome independent of measured tumor burden, with lymphoma and ovarian cancer showing higher pleurodesis rates and gastrointestinal cancer lower rates despite only minor differences in visceral pleural tumor burden across groups [8,17].

Our own tumor-specific analyses likewise showed marked differences in both inflammatory phenotype and catheter positioning. Thoracic malignancies were characterized by higher NLR and a higher frequency of catheter malposition than several other diagnostic categories. These findings suggest that primary tumor type may act as an upstream clinical correlate of the mechanical phenotype; however, the present study cannot distinguish tumor-specific biological effects from differences in sex distribution, pleural disease extent, or other unmeasured characteristics.

This tumor-specific pattern is compatible with either explanation, and our design cannot fully separate a tumor-biology-specific inflammatory effect from the mechanical pathway we identified; this should be regarded as a boundary condition of our mediation model rather than evidence against it.

### 4.5. The Comorbidity–Mortality Discrepancy

An unexpected finding was that 30-day mortality was higher among patients with no indexed comorbidity (25.0%) than among those with two or more comorbidities (7.1%, *p* = 0.19). Importantly, this inverse association was not accompanied by systematic differences in tube caliber, catheter position, or administration method. Instead, patients without indexed comorbidities were substantially younger and had lower serum albumin levels. This combination raises the possibility that conventional comorbidity counts incompletely capture disease severity in MPE and that oncological burden and cancer-associated nutritional depletion may exert greater influence on short-term outcome. Subgroup analysis attributed this to confounding by age and tumor biology rather than to comorbidity itself: comorbidity-free patients who died early were disproportionately younger (<60 years), more inflamed (NLR 13.24 ± 15.06), more eosinopenic, and more cachectic (albumin 28.14 ± 4.54 g/L) than their comorbid counterparts, consistent with aggressive, rapidly progressive malignancy overwhelming an otherwise “healthy” host rather than reflecting protection conferred by fewer comorbidities. This pattern reinforces the broader interpretation that, in MPE, short-term survival is governed less by chronic comorbid burden than by tumor aggressiveness and its downstream nutritional and immunologic consequences.

Interestingly, increasing chronological age was not accompanied by progressively worse pleurodesis outcomes in our cohort. Younger patients showed greater cytology positivity and reintervention rates despite a lower comorbidity burden, suggesting that chronological age and chronic disease burden may not adequately capture the biological severity of malignant pleural disease.

The mortality phenotype was also characterized by lower albumin, higher NLR, neutrophilia, lymphopenia, and eosinopenia. These findings may reflect advanced cancer-associated nutritional depletion and systemic physiological stress; however, neither cachexia severity, tumor burden, disease extent, nor baseline performance status (e.g., ECOG or Karnofsky score) was directly quantified in the present study; unmeasured functional impairment and undocumented disease burden may have independently influenced both treatment selection (e.g., candidacy for VATS versus bedside slurry) and overall survival. Thus, the apparent comorbidity–mortality discrepancy should be interpreted cautiously as a potential consequence of residual, unmeasured differences in oncological disease burden rather than as evidence that comorbidity is protective.

### 4.6. Clinical Implications

These findings support a mechanically oriented, risk-stratified approach to pleurodesis in MPE. Patients with radiographically confirmed non-expandable lung should not be subjected to talc instillation, given the disproportionate failure and mortality risk in this group, and are better served by primary indwelling pleural catheter placement [1,18]. Where drainage is feasible, our propensity-matched analyses provide preliminary support for prioritizing large-bore (≥20 Fr) rigid catheters and, where resources allow, VATS talc poudrage over bedside slurry, particularly in patients with thoracic malignancy or elevated NLR, in whom fibrinous loculation and malposition risk are highest; given the observational design, this should be interpreted as hypothesis-generating for high-risk subgroups rather than a definitive, generalizable treatment recommendation. This is a more selective conclusion than that of the TIME1 trial, which found no difference in pain or overall efficacy between small- and large-bore tubes in a general MPE population [4]; the discrepancy may reflect our cohort’s causal-inference framework and its focus on malposition as an intermediate mechanism rather than on tube caliber as a direct comparator, and it should be interpreted as hypothesis-generating for high-risk subgroups rather than as a categorical departure from BTS/TIME1 guidance.

Similarly, the weighted VATS analysis showed shorter hospitalization and more favorable catheter positioning after adjustment for measured covariates; however, because VATS was inseparably associated with large-bore tube use and optimal catheter position in our cohort, this finding should be considered exploratory and cannot be attributed specifically to the VATS technique independent of tube caliber. These findings support consideration of thoracoscopic management in appropriately selected patients but do not establish independent superiority over bedside slurry.

## 5. Strengths

This study’s principal strength lies in its analytic architecture: multivariable logistic regression with nomogram construction was combined with two independent confounder-adjustment strategies (1:1 PSM and IPTW) and a bootstrapped SEM mediation model, allowing us to move beyond univariate correlation and evaluate a prespecified mediation hypothesis—that inflammation acts on recurrence indirectly, through mechanical barriers—that has not, to our knowledge, been formally modeled in this population before.

## 6. Limitations

Several limitations warrant consideration. First, catheter position and lung re-expansion were assessed only once, at 24–48 h post-procedure, by surgeon consensus rather than independent dual scoring, precluding a formal interobserver agreement statistic; later displacement occurring during the remainder of the 90-day follow-up was not systematically re-imaged and could not be distinguished from de novo recurrence, which may lead to underestimation of late mechanical failure as a distinct contributor. In addition, our binary optimal/suboptimal classification, anchored to placement in the most gravity-dependent pleural recess, may be less applicable in loculated or septated effusions, where the anatomically dependent position may not correspond to the fluid-containing compartment; loculation status was not systematically stratified in this analysis. Second, recurrence and mortality were analyzed as fixed 90-day and 30-day binary endpoints using multivariable logistic and quasi-binomial regression rather than through time-to-event (Kaplan–Meier/Cox) methods; this choice was made because chemical pleurodesis is a single palliative intervention evaluated against two clinically fixed follow-up horizons rather than a continuously accruing hazard, and model discrimination was correspondingly strong (C-index 0.851 for recurrence, 0.825 for mortality), but it precludes direct hazard-ratio comparison with time-to-event literature. Third, because early death competes with the opportunity to observe recurrence, and because the cohort’s overall 30-day mortality (16.4%, rising to 35.6% among patients with inadequate re-expansion) is non-trivial, our recurrence estimates may be subject to competing-risks bias; future multicenter prospective series should apply Fine-Gray subdistribution hazard models to jointly partition the cumulative incidence of recurrence and death [19]. Fourth, chest tube caliber selection and catheter-optimization success varied significantly by attending surgeon, raising the possibility of operator-level clustering; while patient-level covariates (age, sex, diagnosis, inflammatory burden, surgeon case volume) were balanced across exposure groups via PSM and IPTW (all standardized mean differences < 0.10), this does not fully substitute for a hierarchical mixed-effects model, which was not undertaken here. The VATS and slurry groups also differed substantially at baseline, with patients undergoing VATS showing higher albumin and hemoglobin levels and lower inflammatory burden. These differences are compatible with confounding by indication, whereby patients considered fitter for operative management may have been preferentially selected for thoracoscopy. Although IPTW substantially improved balance in measured covariates, residual confounding by unmeasured factors such as performance status cannot be excluded. Fifth, the administration-method comparison was markedly imbalanced (14 VATS poudrage vs. 126 talc slurry cases); although IPTW achieved adequate pseudo-randomization on measured covariates, the small VATS arm limited statistical power for secondary endpoints such as recurrence, whose favorable point estimate (OR = 0.246) did not reach significance (*p* = 0.2057), and this should be interpreted as a type II error risk rather than as evidence of no effect. Furthermore, because all 14 VATS poudrage cases also had large-bore tubes and optimal catheter positioning, the effect of administration method cannot be statistically separated from that of tube caliber and catheter position in this cohort; the extreme effect estimate for catheter placement in the IPTW model (OR < 0.001) reflects this near-complete overlap rather than a precisely estimable independent VATS effect. The VATS-associated findings should therefore be interpreted as exploratory and hypothesis-generating rather than as evidence of an independent causal effect of the VATS technique itself. Finally, as in any retrospective single-center cohort, unmeasured confounders—pleural fluid pH, LDH, glucose, and cytokine levels, effusion volume, and degree of septation/loculation, ECOG performance status, the mechanical compliance of intrapleural adhesions, and systemic antitumor therapy receipt or changes in oncological treatment during the 90-day follow-up period—were not systematically captured and cannot be excluded; therefore, the absence of an independent association between baseline NLR and recurrence in our model does not exclude a broader biological role for local pleural inflammation and concurrent oncological treatment in pleurodesis outcomes, and further prospective studies incorporating serial systemic and local pleural inflammatory parameters, alongside structured treatment data, are needed to clarify this relationship. However, an E-value analysis [20] indicates that an unmeasured confounder would need to be associated with both the exposure and the outcome by a risk ratio of approximately 9- to 10-fold to fully explain away our largest effect estimate (trapped lung, aOR = 9.89), a magnitude for which there is no obvious biological candidate in this clinical context. Our procedural complication endpoint was restricted to major complications requiring additional intervention; minor, self-limited post-procedural effects were not systematically abstracted from retrospective records, and only one major complication (ARDS) occurred in the entire cohort, with no cases of severe hemorrhage, pneumonia, or empyema meeting this threshold—this should be interpreted as reflecting our narrow complication definition and the safety profile of the major-complication category specifically, not the absence of any post-procedural morbidity. The exact breakdown of patients diagnosed by pleural fluid cytology alone versus pleural biopsy was not separately tabulated, and pleural effusion volume was not systematically quantified, as these are not reliably captured in retrospective chart review; both should be prospectively recorded in future studies.

## 7. Conclusions

In the present model, baseline Neutrophil-to-Lymphocyte Ratio (NLR) was not an independent predictor of pleurodesis recurrence after adjustment for mechanical factors—specifically, inadequate lung re-expansion and suboptimal catheter position, the strongest independent predictors identified. Exploratory mediation analysis suggested a possible indirect pathway by which systemic inflammation could contribute to recurrence through these mechanical intermediates, but this should be regarded as hypothesis-generating and does not exclude a broader role for local pleural inflammatory processes—pleural fluid pH, LDH, glucose, cytokine levels, effusion volume, and degree of septation or loculation—which were not systematically measured in this study. Clinicians managing MPE should consider mechanical optimization—large-bore drainage, VATS poudrage where feasible, and early transition to indwelling pleural catheters in patients with non-expandable lung or severe cachexia—as an important, evidence-supported component of risk stratification, alongside rather than in place of inflammation-based markers, pending prospective confirmation of the mechanisms proposed here.

## Figures and Tables

**Table 1:** Baseline Demographic, Procedural, Biochemical, and Clinical Characteristics of the Cohort (*N* = 140).

Characteristic	Value (*N* = 140)
***Demographic Variables***	
Age (years), Mean ± SD	62.23 ± 13.14
Sex—Male, *n* (%)	77 (55.0%)
Sex—Female, *n* (%)	63 (45.0%)
Age Group—<65/≥65 years	79 (56.4%)/61 (43.6%)
Age Group—<60/60–74/≥75 years	54 (38.6%)/60 (42.9%)/26 (18.6%)
Laterality—Right/Left	85 (60.7%)/55 (39.3%)
Comorbidity Burden—None/Single/Multiple (≥2)	72 (51.4%)/26 (18.6%)/42 (30.0%)
Indexed Comorbidity Count (n), Mean ± SD	0.96 ± 1.23
***Biochemical and Inflammatory Biomarkers, Mean ± SD***	
C-Reactive Protein (mg/L)	74.52 ± 74.03
Serum Albumin (g/L)	30.81 ± 6.74
Hemoglobin (g/dL)	11.03 ± 1.83
Neutrophil-to-Lymphocyte Ratio (NLR)	9.51 ± 11.13
CRP/Albumin Ratio (CAR)	2.65 ± 2.92
***Diagnostic Classification***	
Primary Diagnosis—Thoracic Malignancy	58 (41.4%)
Primary Diagnosis—Thoracic Malignancy: Adenocarcinoma (lung-predominant)	41 (29.3%)
Primary Diagnosis—Thoracic Malignancy: Squamous Cell Carcinoma	9 (6.4%)
Primary Diagnosis—Thoracic Malignancy: Small Cell/Neuroendocrine Carcinoma	8 (5.7%)
Primary Diagnosis—Breast Carcinoma	22 (15.7%)
Primary Diagnosis—GI/Gynecological Malignancy	31 (22.1%)
Primary Diagnosis—Other Malignancy	22 (15.7%)
Primary Diagnosis—Benign Effusion ^a^	7 (5.0%)
***Procedural and Mechanical Parameters***	
Tube Caliber—Large-bore (≥20 Fr)	80 (57.1%)
Tube Caliber—Small-bore (≤16 Fr)	60 (42.9%)
Administration Method—Talc Slurry	126 (90.0%)
Administration Method—VATS Talc Poudrage	14 (10.0%)
Catheter Position—Optimal	58 (41.4%)
Catheter Position—Suboptimal (Malposition)	82 (58.6%)
Lung Re-expansion—Adequate	95 (67.9%)
Lung Re-expansion—Inadequate (NEL/Trapped Lung)	45 (32.1%)
***Clinical Endpoints***	
Hospital Length of Stay (days), Mean ± SD	13.51 ± 10.04
Pleurodesis Recurrence	40 (28.6%)
Reintervention Requirement	32 (22.9%)
30-Day All-Cause Mortality	23 (16.4%)
Procedural Complication—ARDS	1 (0.7%)

**Note.** Continuous variables are presented as mean ± standard deviation (Mean ± SD); categorical variables are presented as frequency and percentage (*n*, %). As all patients received talc administration (poudrage or slurry) per standard protocol, no variance was observed for this parameter. For binary clinical endpoints, only the row representing the event is reported. ^a^ Seven patients with benign, non-MPE (parapneumonic, tuberculous, or other non-malignant etiologies) were intentionally retained in the cohort as a comparator subset for etiological polarization analysis, per the exclusion criteria detailed in Section 2.

**Table 2:** Statistically Significant Comparative Findings Between Pleurodesis Recurrence Groups (*p* < 0.05).

Characteristic	No Recurrence (*n* = 100)	Recurrence (*n* = 40)	Test Statistic, *p*-Value
***Continuous Variables, Mean ± SD***			
Chest Tube Diameter (Fr)	24.32 ± 9.11	21.02 ± 9.72	U = 2394.0, *p* = 0.48
Hospital Length of Stay (days)	12.07 ± 8.79	17.12 ± 12.02	U = 1265.0, *p* < 0.01
***Categorical Variables, n (%)***			
Catheter Position—Optimal	53 (53.0%)	5 (12.5%)	χ^2^(1) = 17.680, *p* < 0.01
Catheter Position—Suboptimal	47 (47.0%)	35 (87.5%)	
Lung Re-expansion—Adequate	84 (84.0%)	11 (27.5%)	χ^2^(1) = 39.266, *p* < 0.01
Lung Re-expansion—Inadequate	16 (16.0%)	29 (72.5%)	
Reintervention Requirement	0 (0.0%)	32 (80.0%)	χ^2^(1) = 99.216, *p* < 0.01
30-Day All-Cause Mortality	9 (9.0%)	14 (35.0%)	χ^2^(1) = 12.238, *p* < 0.01

**Note.** Continuous variables were compared using the Mann–Whitney U test; categorical variables were compared using Pearson’s chi-square test. Only variables reaching statistical significance (*p* < 0.05) are shown; the recurrence outcome itself and its directly dependent subcategories were excluded to avoid autocorrelation.

**Table 3:** Statistically Significant Comparative Findings Between Catheter Position Groups (*p* < 0.05).

Characteristic	Optimal (*n* = 58)	Suboptimal (*n* = 82)	Test Statistic, *p*-Value
***Continuous Variables, Mean ± SD***			
Chest Tube Diameter (Fr)	28.90 ± 6.95	19.48 ± 8.91	U = 3611.5, *p* < 0.01
Neutrophil Percentage (%)	73.56 ± 11.50	77.31 ± 12.39	U = 1848.5, *p* = 0.25
Lymphocyte Percentage (%)	16.62 ± 9.01	13.07 ± 10.40	U = 3094.5, *p* = 0.02
Neutrophil-to-Lymphocyte Ratio (NLR)	7.24 ± 8.11	11.11 ± 12.65	U = 1705.0, *p* = 0.04
Hospital Length of Stay (days)	12.86 ± 11.80	13.98 ± 8.63	U = 1745.5, *p* = 0.07
***Categorical Variables, n (%)***			
Tube Caliber—Large-bore (≥20 Fr)	50 (86.2%)	30 (36.6%)	χ^2^(1) = 32.160, *p* < 0.01
Tube Caliber—Small-bore (≤16 Fr)	8 (13.8%)	52 (63.4%)	
Administration Method—Slurry	44 (75.9%)	82 (100.0%)	χ^2^(1) = 19.392, *p* < 0.01
Administration Method—VATS Poudrage	14 (24.1%)	0 (0.0%)	
Lung Re-expansion—Adequate	51 (87.9%)	44 (53.7%)	χ^2^(1) = 16.757, *p* < 0.01
Lung Re-expansion—Inadequate	7 (12.1%)	38 (46.3%)	
Pleurodesis Recurrence	5 (8.6%)	35 (42.7%)	χ^2^(1) = 17.680, *p* < 0.01
Reintervention Requirement	4 (6.9%)	28 (34.1%)	χ^2^(1) = 12.802, *p* < 0.01
30-Day All-Cause Mortality	4 (6.9%)	19 (23.2%)	χ^2^(1) = 5.421, *p* = 0.11

**Note.** Continuous variables were compared using the Mann–Whitney U test; categorical variables were compared using Pearson’s chi-square test. Only variables reaching statistical significance (*p* < 0.05) are shown; catheter position itself was excluded to avoid autocorrelation.

**Table 4:** Statistically Significant Comparative Findings Between Lung Re-expansion Groups (*p* < 0.05).

Characteristic	Adequate Re-Expansion (*n* = 95)	Inadequate Re-Expansion (*n* = 45)	Test Statistic, *p*-Value
***Continuous Variables, Mean ± SD***			
C-Reactive Protein (mg/L)	63.76 ± 65.45	97.24 ± 85.92	U = 1599.5, *p* = 0.17
Absolute Neutrophil Count (/µL)	6435.82 ± 4512.05	7792.22 ± 4006.29	U = 1696.0, *p* = 0.49
CRP/Albumin Ratio (CAR)	2.25 ± 2.54	3.50 ± 3.48	U = 1652.0, *p* = 0.31
Hospital Length of Stay (days)	12.17 ± 9.09	16.36 ± 11.39	U = 1423.0, *p* = 0.01
***Categorical Variables, n (%)***			
Sex—Female	52 (54.7%)	11 (24.4%)	χ^2^(1) = 10.131, *p* = 0.01
Sex—Male	43 (45.3%)	34 (75.6%)	
Catheter Position—Optimal	51 (53.7%)	7 (15.6%)	χ^2^(1) = 16.757, *p* < 0.01
Catheter Position—Suboptimal	44 (46.3%)	38 (84.4%)	
Pleurodesis Recurrence	11 (11.6%)	29 (64.4%)	χ^2^(1) = 39.266, *p* < 0.01
Reintervention Requirement	9 (9.5%)	23 (51.1%)	χ^2^(1) = 27.708, *p* < 0.01
30-Day All-Cause Mortality	7 (7.4%)	16 (35.6%)	χ^2^(1) = 15.677, *p* < 0.01

**Note.** Continuous variables were compared using the Mann–Whitney U test; categorical variables were compared using Pearson’s chi-square test. Only variables reaching statistical significance (*p* < 0.05) are shown; lung re-expansion status itself was excluded to avoid autocorrelation.

**Table 5:** Statistically Significant Comparative Findings Between 30-Day Mortality Groups (*p* < 0.05).

Characteristic	Alive at 30 Days (*n* = 117)	Deceased at 30 Days (*n* = 23)	Test Statistic, *p*-Value
***Continuous Variables, Mean ± SD***			
Serum Albumin (g/L)	31.33 ± 6.99	28.14 ± 4.54	t(45.43) = 2.785, *p* = 0.08
Neutrophil-to-Lymphocyte Ratio (NLR)	8.78 ± 10.11	13.24 ± 15.06	U = 954.0, *p* = 0.28
Neutrophil Percentage (%)	74.82 ± 11.88	80.50 ± 12.54	U = 898.5, *p* = 0.12
Lymphocyte Percentage (%)	15.06 ± 9.60	11.89 ± 11.51	U = 1717.0, *p* = 0.37
Eosinophil Percentage (%)	1.82 ± 1.91	0.93 ± 1.12	U = 1739.5, *p* = 0.27
Indexed Comorbidity Count (n)	1.03 ± 1.17	0.61 ± 1.50	U = 1750.5, *p* = 0.14
Hospital Length of Stay (days)	11.99 ± 9.35	21.26 ± 10.05	U = 575.0, *p* < 0.01
***Categorical Variables, n (%)***			
Catheter Position—Optimal	54 (46.2%)	4 (17.4%)	χ^2^(1) = 5.421, *p* = 0.20
Catheter Position—Suboptimal	63 (53.8%)	19 (82.6%)	
Lung Re-expansion—Adequate	88 (75.2%)	7 (30.4%)	χ^2^(1) = 15.677, *p* < 0.01
Lung Re-expansion—Inadequate	29 (24.8%)	16 (69.6%)	
Comorbidity Burden—None	54 (46.2%)	18 (78.3%)	Fisher’s *p* = 0.26
Comorbidity Burden—Single/Multiple	24 (20.5%)/39 (33.3%)	2 (8.7%)/3 (13.0%)	
Pleurodesis Recurrence	26 (22.2%)	14 (60.9%)	χ^2^(1) = 12.238, *p* < 0.01
Reintervention Requirement	21 (17.9%)	11 (47.8%)	χ^2^(1) = 8.110, *p* = 0.04

**Note.** Continuous variables were compared using the Mann–Whitney U test, except serum albumin (independent-samples *t*-test with Welch correction); categorical variables were compared using Pearson’s chi-square test or Fisher’s exact test where expected cell counts were <5. Only variables reaching statistical significance (*p* < 0.05) are shown; 30-day mortality itself was excluded to avoid autocorrelation.

**Table 6:** Multivariable Logistic Regression Models for Pleurodesis Recurrence, 30-Day Mortality, and Non-Expandable Lung.

Predictor	aOR	95% CI	*p*-Value
**Model 1: Pleurodesis Recurrence (ROC-AUC = 0.851)**			
Lung Expansion—Inadequate	9.89	3.98–26.29	<0.0001
Catheter Position—Suboptimal	3.80	1.11–14.84	0.0407
Chest Tube—Small-bore	1.25	0.43–3.63	0.6779
NLR	1.01	0.97–1.05	0.6794
Age (years)	0.97	0.94–1.01	0.1586
**Model 2: 30-Day Mortality (ROC-AUC = 0.825)**			
Lung Expansion—Inadequate	5.21	1.77–16.74	0.0036
Catheter Position—Suboptimal	1.82	0.51–7.45	0.3714
Serum Albumin (g/L)	0.93	0.85–1.01	0.0893
Eosinophil Percentage (%)	0.73	0.45–1.06	0.1521
Indexed Comorbidity Count	0.80	0.47–1.23	0.3488
NLR	1.01	0.97–1.06	0.5297
**Model 3: Non-Expandable Lung (Trapped Lung)**			
Sex—Male	3.67	1.65–8.70	0.0020
Chest Tube—Small-bore	1.48	0.69–3.22	0.3137
CRP/Albumin Ratio (CAR)	1.07	0.64–1.79	0.7982
C-Reactive Protein (mg/L)	1.00	0.98–1.02	0.7273
NLR	1.00	0.96–1.03	0.9471

**Note.** aOR = adjusted odds ratio, reflecting the independent effect of each predictor with all other covariates in the model held constant. Reference categories: Optimal (Catheter Position), Adequate/Complete Visceroparietal Apposition (Lung Expansion), Large-bore ≥ 20 Fr (Chest Tube), Female (Sex).

**Table 7:** IPTW and PSM Confounder-Adjusted Pseudo-Cohort Outcome Analyses.

Clinical Outcome	Effect Estimate	Method	*p*-Value
**Model 1: IPTW-Weighted Cohort (VATS Poudrage vs. Talc Slurry, ATT)**			
Hospital Length of Stay	−6.40 days	Weighted linear regression	0.0074
Suboptimal Catheter Placement	OR < 0.001	Weighted quasi-binomial regression	<0.0001
Pleurodesis Recurrence	OR = 0.246	Weighted quasi-binomial regression	0.2057
**Model 2: 1:1 PSM-Matched Cohort (Large-Bore vs. Small-Bore Tube, *N* = 98 pairs)**			
Suboptimal Catheter Placement	Matched OR = 0.096	Matched binomial regression	<0.0001
Pleurodesis Recurrence	Matched OR = 0.405	Matched binomial regression	0.0493
Non-Expandable Lung	Matched OR = 0.457	Matched binomial regression	0.0824

**Note.** ATT = average treatment effect on the treated. Baseline confounders in both models: age, sex, albumin, hemoglobin, NLR, comorbidity burden, and primary diagnosis category. Covariate balance was confirmed via Love plots (SMD < 0.10 threshold; Supplementary Figures S3 and S4).

## Data Availability

The data presented in this study are available on request from the corresponding author, due to patient privacy and institutional ethics restrictions.

## Supplementary Materials

The following supporting information can be downloaded at: https://www.mdpi.com/article/10.3390/medicina62091763/s1, Figure S1: Clinical nomogram for bedside prediction of individualized pleurodesis recurrence probability, based on catheter optimality, lung re-expansion status, chest tube caliber, NLR, and age; Figure S2: Clinical nomogram for bedside prediction of individualized 30-day mortality probability, based on serum albumin, NLR, eosinophil percentage, catheter optimality, and lung re-expansion status; Figure S3: Covariate balance (Love plot) before and after IPTW for administration method (VATS poudrage vs. talc slurry); Figure S4: Covariate balance (Love plot) before and after 1:1 propensity score matching for chest tube caliber (large-bore vs. small-bore); Figure S5: Structural equation model path diagram illustrating the mechanical mediation of systemic inflammation (NLR) on pleurodesis recurrence via trapped lung and catheter malposition; Table S1: Structural Equation Modeling Mediation Analysis: Direct, Indirect, and Total Effects of Systemic Inflammation (NLR) on Pleurodesis Recurrence via Mechanical Barriers (5000-Bootstrap BCa Simulation); Table S2: Sensitivity Analysis: Multivariable Logistic Regression Models for Pleurodesis Recurrence and 30-Day Mortality in the Strictly Malignant Cohort (Benign Effusions Excluded, *N* = 133).
