# Supplementary material for: Determinants of Chemical Pleurodesis Recurrence in Malignant Pleural Effusion: Mechanical Apposition or Systemic Inflammation? A Retrospective Cohort Study
Source: Medicina (Kaunas). 2026 Sep 13;62(9):1763. doi: 10.3390/medicina62091763 (PMC13608950; doi:10.3390/medicina62091763)
Supplement: Supplementary file 1 [file medicina-62-01763-s001.zip › medicina-4532255-supplementary.pdf]

Supplementary Materials

Determinants of Chemical Pleurodesis Recurrence in Malignant Pleural Effusion: Mechanical Apposition or Systemic Inflammation? A Retrospective Cohort Study

Caner İşevi, Mehmet Gökhan Pirzirenli, Eren Yılmaz, Yavuz Karaca, Burçin Çelik, Yasemin Büyükkarabacak

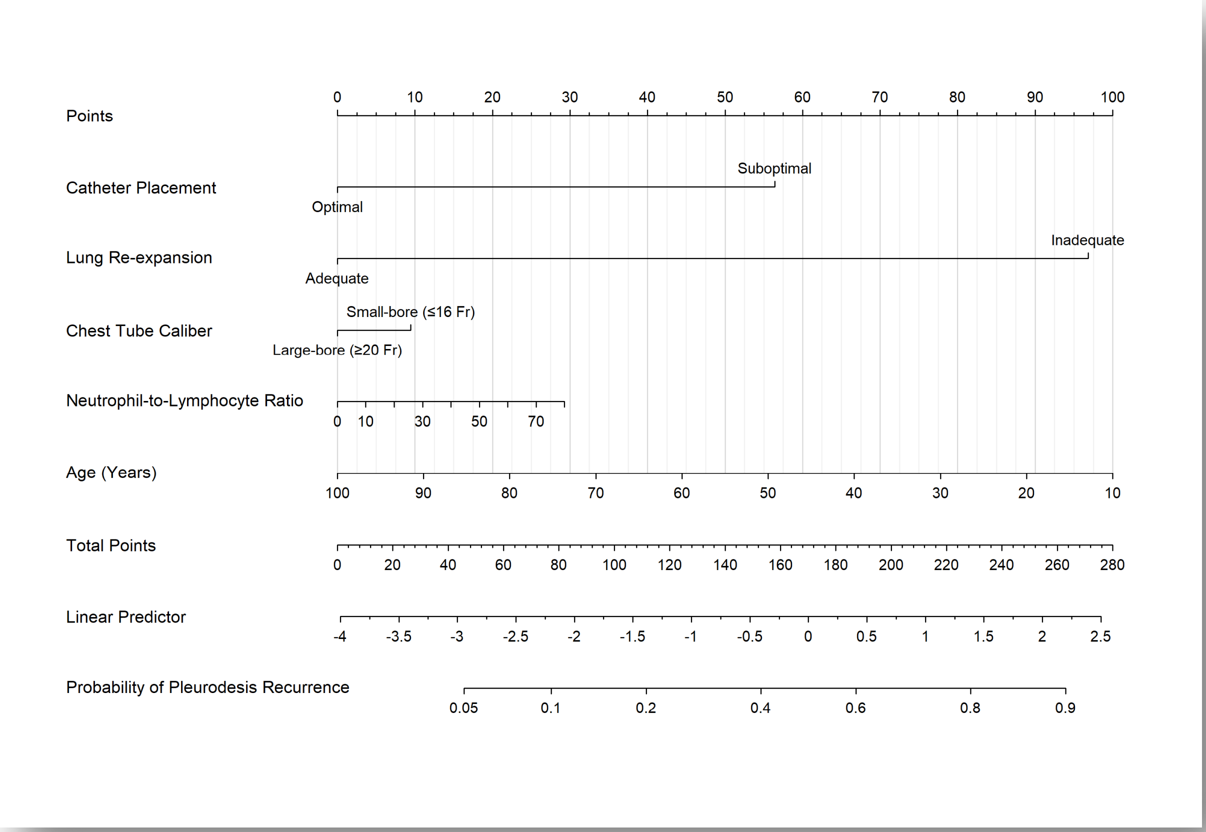

Supplementary Figure S1. *Clinical nomogram for bedside prediction of individualized pleurodesis recurrence probability, based on catheter optimality, lung re-expansion status, chest tube caliber, NLR, and age.*

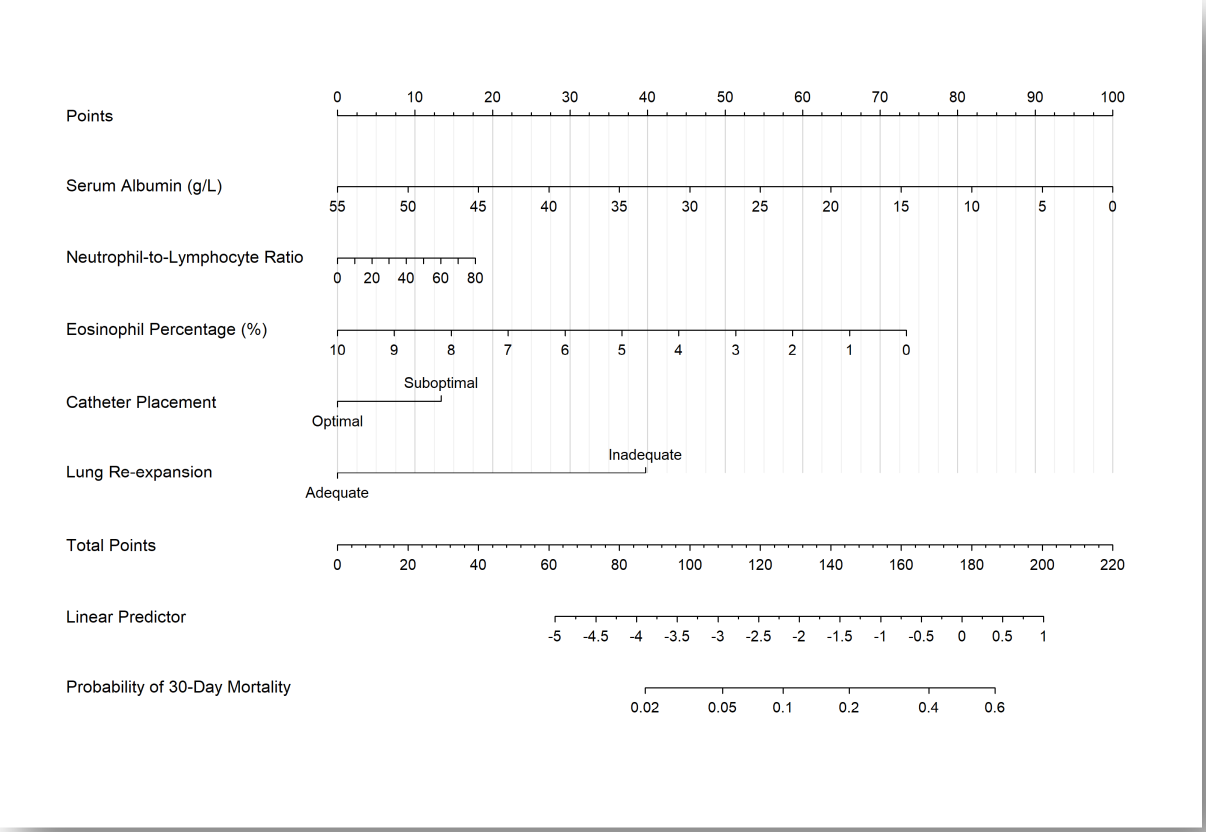

Supplementary Figure S2. *Clinical nomogram for bedside prediction of individualized 30-day mortality probability, based on serum albumin, NLR, eosinophil percentage, catheter optimality, and lung re-expansion status.*

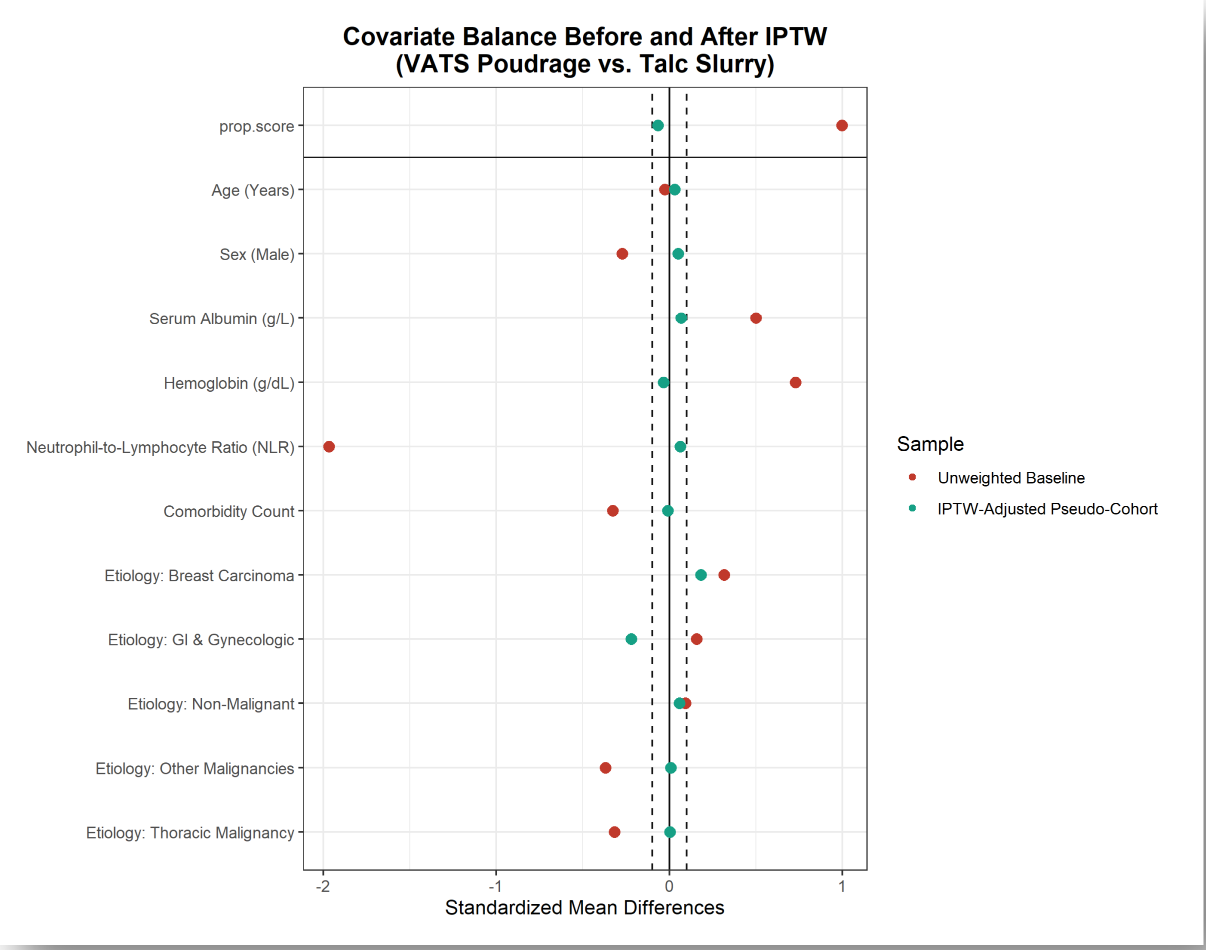

Supplementary Figure S3. *Covariate balance (Love plot) before and after IPTW weighting for administration method (VATS poudrage vs. talc slurry).*

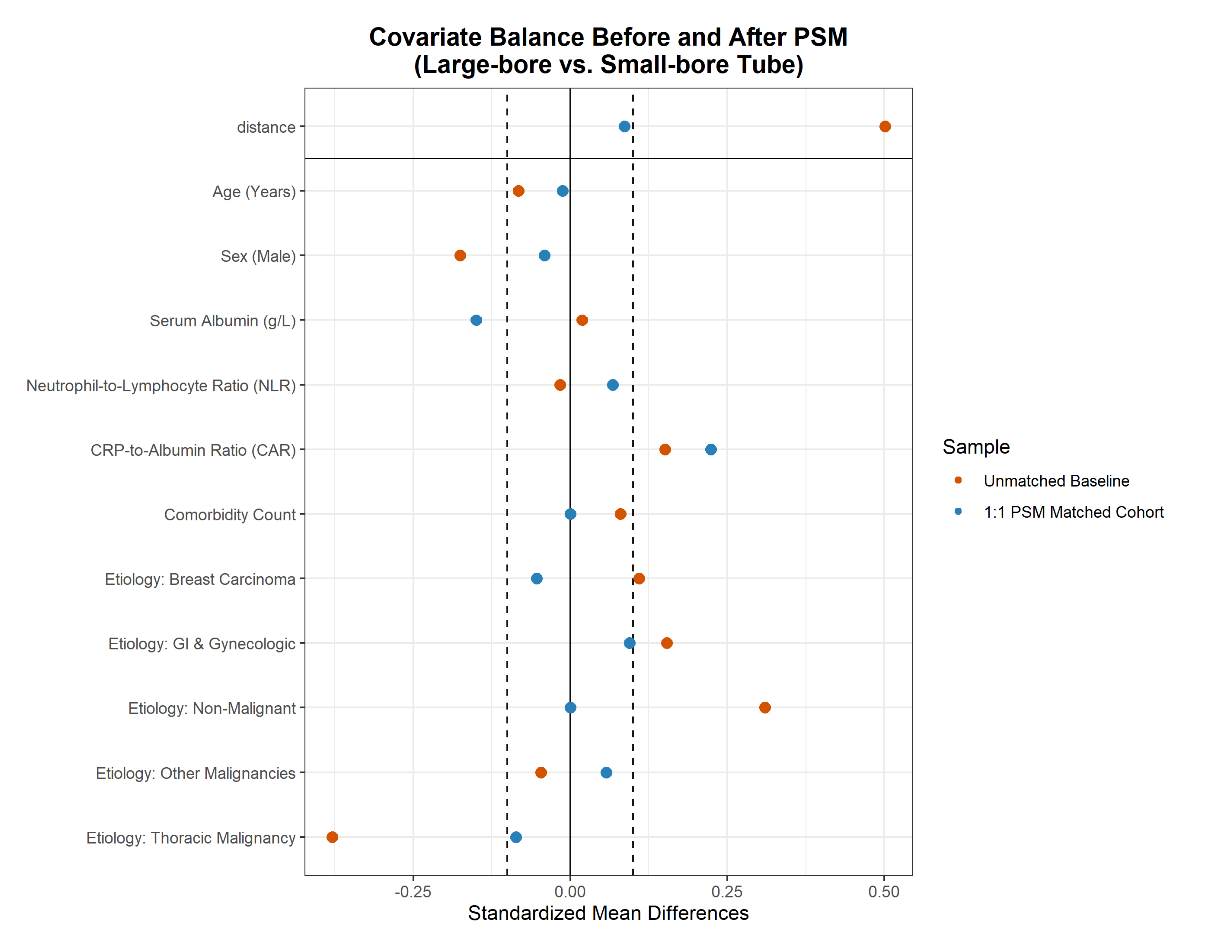

Supplementary Figure S4. *Covariate balance (Love plot) before and after 1:1 propensity score matching for chest tube caliber (large-bore vs. small-bore).*

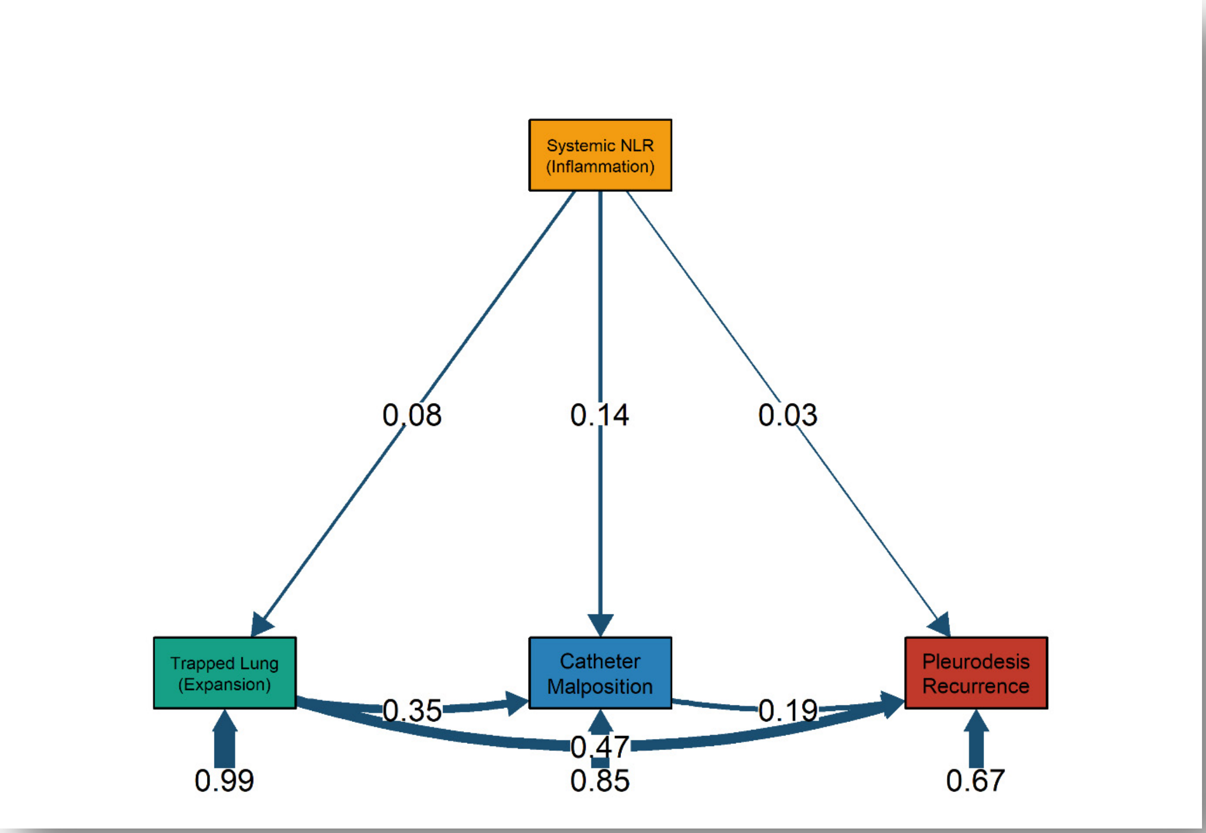

Supplementary Figure S5. *Structural equation model path diagram illustrating the mechanical mediation of systemic inflammation (NLR) on pleurodesis recurrence via trapped lung and catheter malposition.*

Supplementary Table S1: *Structural Equation Modeling Mediation Analysis: Direct, Indirect, and Total Effects of Systemic Inflammation (NLR) on Pleurodesis Recurrence via Mechanical Barriers (5000-Bootstrap BCa Simulation)*

Effect Type	Structural Pathway	β	95% Bootstrap CI	p
**Direct**	NLR → Recurrence	0.0147	−0.0472 to 0.0615	.5897
**Indirect 1**	NLR → Inadequate Re-expansion → Recurrence	0.0178	−0.0224 to 0.0550	.3621
**Indirect 2**	NLR → Catheter Malposition → Recurrence	0.0126	0.0014 to 0.0416	.1961
**Serial Mediation**	NLR → Inadequate Re-expansion → Malposition → Recurrence	0.0026	−0.0023 to 0.0110	.4164
**Total Indirect**	Cumulative sum of all mediating pathways	0.0330	−0.0155 to 0.0742	.1389
**Total Combined**	Direct + Total Indirect Effect	0.0476	−0.0409 to 0.1142	.2287

Note. *NLR was standardized (z-score) prior to model estimation. Bias-corrected and accelerated (BCa) bootstrap confidence intervals (5000 replications) are the primary inferential criterion for mediation*

effects; a CI excluding zero indicates a statistically supported pathway irrespective of the corresponding normal-approximation p-value.

Supplementary Table S2: *Sensitivity Analysis: Multivariable Logistic Regression Models for Pleurodesis Recurrence and 30-Day Mortality in the Strictly Malignant Cohort (Benign Effusions Excluded, N=133)*

Predictor	Adjusted Odds Ratio (aOR)	95% CI	p-value
**Model 1: Pleurodesis Recurrence**			
Lung Expansion — Inadequate	9.65	3.81 – 26.10	< .0001
Catheter Position — Suboptimal	4.43	1.22 – 19.20	.0315
Chest Tube — Small-bore (≤ 16 Fr)	1.07	0.36 – 3.12	.9010
NLR	1.01	0.96 – 1.06	.6410
Age (years)	0.98	0.94 – 1.02	.3430
**Model 2: 30-Day Mortality**			
Lung Expansion — Inadequate	4.49	1.48 – 14.80	.0098
Catheter Position — Suboptimal	2.52	0.64 – 12.60	.2070
Serum Albumin (g/L)	0.91	0.83 – 1.00	.0614
Eosinophil Percentage (%)	0.70	0.41 – 1.03	.1210
Indexed Comorbidity Count	0.90	0.53 – 1.40	.6860
NLR	1.01	0.97 – 1.06	.5220
